# Heme oxygenase 1 activity mediates red blood cell clearance and tail fin regeneration in zebrafish larvae

**DOI:** 10.1038/s41598-026-54996-x

**Published:** 2026-06-02

**Authors:** Ashmair Mirza, Scott Crawte, Carlos Muñoz-Montecinos, Shritama Mukherjee, Graham J. Lieschke, Miguel L. Allende, Rodrigo A. Morales Castro

**Affiliations:** 1 Division of Clinical Immunology, Department of Laboratory Medicine (Labmed), Karolinska Institutet, 14152 Huddinge, Sweden; 2 Departamento de Biología, Facultad de Ciencias, Universidad de Chile, 7800003 Ñuñoa, Santiago, Chile; 3 Australian Regenerative Medicine Institute (ARMI), Monash University, Clayton, Victoria 3800 Australia; 4 Millennium Institute Center for Genome Regulation, Santiago, Chile

**Keywords:** Biochemistry, Biological techniques, Biotechnology, Cell biology, Molecular biology

## Abstract

Tissue injury triggers a tightly regulated cascade of events that transition from inflammation to resolution and ultimately tissue remodeling. Although the cellular dynamics of immune cells during these phases are increasingly well-characterized, the molecular mediators orchestrating the response to injury are yet to be fully elucidated. Based on a zebrafish model of tissue injury and using proteomic, *in situ* RNA expression analyses, and novel transgenic fluorescent reporters, we aimed to uncover relevant molecular mediators of tissue inflammation, resolution, and regeneration. We found that red blood cells (RBCs) accumulated at the injury site after tail fin amputation in zebrafish larvae, reaching its peak during the inflammatory phase and decreasing together with the resolution of inflammation. Furthermore, we observed that the heme scavenger and cytoprotective enzyme heme oxygenase 1 (Hmox1) is expressed in the injury site of amputated tail fins, and that macrophages were the main source of the functional *hmox1a* paralog. Pharmacological and morpholino-mediated inhibition of Hmox1 impaired RBC clearance and tail fin regeneration. In addition, depletion of macrophages led to impaired RBC clearance, phenocopying Hmox1 inhibition. Altogether, our findings reveal a novel role for Hmox1 in shaping the regenerative microenvironment and identify RBCs and *hmox1a*-expressing macrophages as previously overlooked players in the zebrafish injury response. This work underscores a new link between heme metabolism, immune regulation, and tissue regeneration *in vivo*.

**Supplementary Information:**

The online version contains supplementary material available at 10.1038/s41598-026-54996-x.

## Introduction

The tissue response to injury is composed of an orchestrated cascade of events, including wound closure, inflammation, proliferation, and tissue remodeling. In non-regenerative systems, these events normally culminate in scar formation, whereas in fully regenerative systems, the outcome is the formation of a tissue that is architecturally and functionally identical to the tissue before injury. Immune cells are key players in the cascade of events following tissue damage. Neutrophils and macrophages are rapidly recruited to the injured tissue and promote local pro-inflammatory responses primarily to protect the tissue from potential pathogenic infection^1,2^. Beyond their pro-inflammatory functions, macrophages are also responsible for clearing apoptotic and damaged cells at the injury site, counteract pro-inflammatory signals, and produce growth factors that initiate wound healing and tissue regeneration^1^. To perform these tasks, macrophages undergo phenotypic and functional changes that endow them with anti-inflammatory and pro-regenerative functions^3^. However, the temporal dynamics of these anti-inflammatory programs in macrophages and their precise contribution to the resolution of inflammation and tissue regeneration remain poorly understood.

Zebrafish (*Danio rerio*) has become a well-established animal model for studying inflammatory responses thanks to its optical transparency during larval stages, which allows intravital and real-time visualization of immune responses following injury. Additionally, its high regenerative capacity has facilitated studying the impact of immune cells during tissue regeneration. The zebrafish larva possesses functional hematopoietic and innate immune cells^4–6^, with the latter being critical orchestrators of tissue inflammation and regeneration. Using the tail fin amputation model in zebrafish larvae, we and others have previously shown that macrophages are the predominant immune cell type contributing to tissue regeneration^7–10^. Mechanistically, recruited macrophages promote the resolution of inflammation by attenuating local production of pro-apoptotic Il1b^11^ and suppressing ROS production^10^, while they activate regenerative programs on stromal cells through Tnfa signaling^9^. However, which anti-inflammatory proteins are expressed by zebrafish macrophages and play a role in the resolution of inflammation and tissue regeneration remains under investigation. Studies from mammals show that expression of the heme scavenger and cytoprotective enzyme heme oxygenase 1 (HMOX1, commonly abbreviated as HO-1) confers macrophages with anti-inflammatory^12^ and pro-regenerative functions^13^. Zebrafish possess two orthologs of the mammalian *HMOX1* gene: *hmox1a* and *hmox1b*. Although *hmox1a* has been associated with macrophage function^14^, it remains unclear whether *hmox1a* is expressed by zebrafish macrophages during injury and plays a role in the resolution of inflammation and tissue regeneration.

In this work, using an unbiased proteomic analysis, we found that red blood cells (RBCs) accumulated at the injury site after tail fin amputation in zebrafish larvae. RBCs, detected by o-dianisidine staining and the *gata1a* transgenic reporter line, peaked during the inflammatory phase and decreased before regeneration took place. By performing RNA expression analysis and using a novel fluorescent transgenic line, we observed the expression of the functional *hmox1a* paralog in macrophages at the injury site of amputated tail fins. Pharmacological inhibition of Hmox1 activity following tail fin amputation and morpholino-mediated *hmox1a* knockdown impaired RBC clearance and tail fin regeneration Furthermore, depletion of macrophages led to impaired RBC clearance from the injury site. Altogether, our findings reveal a novel role for Hmox1 in shaping the regenerative microenvironment and identify RBCs and *hmox1a*-expressing macrophages as relevant players in the zebrafish injury response.

## Results

### Accumulation of red blood cells at the injury site after tail fin amputation

**Fig. 1:**
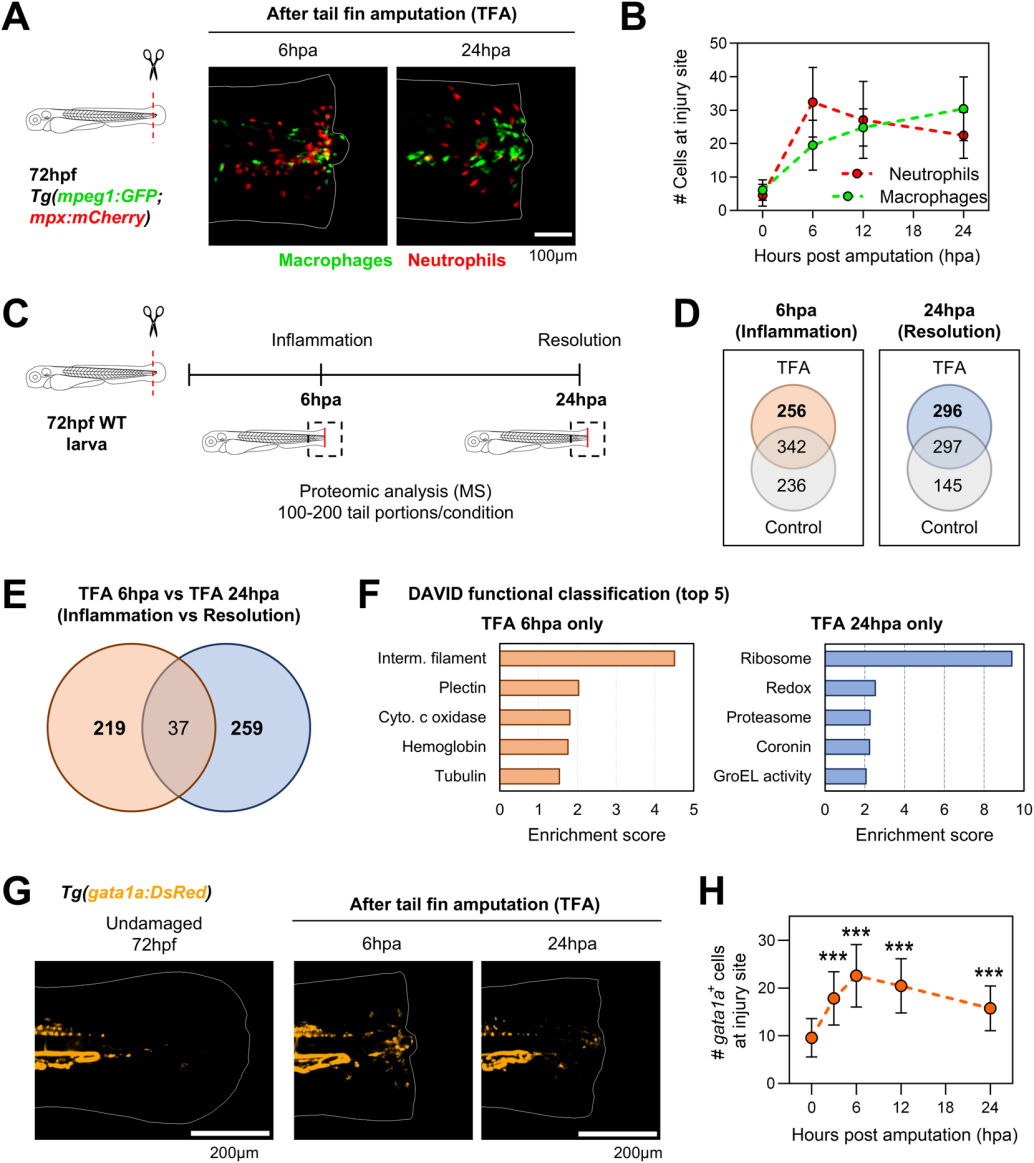
Red blood cells accumulate at the injury site following tail fin amputation in zebrafish larvae. (**A**) Representative images of double transgenic reporter *Tg(mpeg1:GFP; mpx:mCherry)* after tail fin amputation. Scale bar = 100μm. (**B**) Quantification of neutrophils and macrophages after tal fin amputation (mean ± SD; n = 40 larvae per condition/timepoint). (**C**) Schematic for proteomic analysis after tail fin amputation. (**D**) Venn diagram of proteins exclusively identified in amputated larvae versus control during inflammation (6hpa) and resolution (24hpa). The number of identified proteins is indicated in the figure. (**E**) Identification of proteins exclusively expressed during inflammation (6hpa) and resolution (24hpa). Numbers of proteins per group are indicated in the Venn diagram. (**F**) DAVID functional classification of proteins exclusively identified in amputated tail fins during inflammation (6hpa) and resolution (24hpa). The top 5 classifications per studied time point are plotted. (**G**) Representative pictures of control and amputated tail fins of *Tg(gata1a:DsRed)* zebrafish reporter larvae. Scale bar = 200μm. (**H**) Quantification of *gata1a*^+^ cells in the injury site of tail fin amputated larvae at multiple timepoints after injury (mean ± SD; n = 20-30 larvae per timepoint). Statistical comparisons were performed against non-amputated controls (0hpa).One-way ANOVA with Tukey’s post-comparisons were performed in H at the indicated timepoints over control (0hpa). ***p<0.001.

To induce inflammation, we used a previously described model of tail fin amputation in 72 hours post fertilization (hpf) larvae^10^ (Fig. S1A). The injury does not cause damage to the caudal vein loop or any blood vessel near the injury site, as confirmed by amputations performed in the endothelial cell reporter *Tg(fli1:EGFP)* (Fig. S1B). We analyzed the recruitment of neutrophils and macrophages to the injury site in double transgenic reporter *Tg(mpeg1:EGFP; mpx:mCherry)* larvae. In line with previous reports^7,15^, we observed an “inflammation” phase at 6 hours post amputation (hpa), characterized by a peak of neutrophils recruited to the injury site (Fig. 1A-B) and a modest increase in the recruitment of macrophages. After 6hpa, the number of neutrophils at the injury site decreased, while the number of recruited macrophages continued increasing. By 24hpa, we observed a “resolution” phase where macrophages were predominant over neutrophils (Fig. 1A-B). We selected 6hpa (inflammation) and 24hpa (resolution) to perform proteomic analyses of injured tissues and uninjured time-matched controls (Fig. 1C Supplementary Table S1). After filtering and removal of duplicated proteins (see methods), we sought to identify the proteins that were exclusively expressed during inflammation and resolution. By comparing with their respective time-matched controls, we identified 256 proteins that were exclusively present in injured tissues during inflammation, while 296 proteins were detected exclusively in the injured tissue at the resolution phase (Fig. 1D, Supplementary Table S2). Next, we compared the proteins exclusive to injured tissues in inflammation versus resolution, and we found 219 inflammation-specific and 259 resolution-specific proteins (Fig. 1E, Supplementary Table S2). Functional classification analysis of identified phase-specific proteins using DAVID bioinformatic resources^16^ gave us insights into proteins and biological processes that were active during inflammation and resolution (Fig. 1F). Among them, we found targets previously described during resolution and important for tissue regeneration, such as hsp60/GroEL activity^17^ and Coronin, the latter expressed by myeloid cells during tissue damage^7^. During inflammation, we found increased expression of mitochondrial cytochrome c oxidase proteins (Cox5aa and Cox5ab), supporting increased inflammation and suggesting higher mitochondrial activity. Interestingly, we found increased levels of the hemoglobin (Hb) proteins Hbbe2, Ba1 (Hbba1), and Hbbe3 during inflammation (Fig. 1F, Supplementary Table S3). Since these proteins are common markers for erythroid cells, we analyzed the expression of the coding genes for these proteins (*hbbe2*, *hbba1* and *hbbe3*) in a previously published single-cell dataset of the developing zebrafish embryo^18^ (Supplementary Fig. S2A-B). We found that expression of these genes is restricted to red blood cells (RBCs) and are not expressed by immune cell types in homeostasis (Supplementary Fig. S2C). To validate these findings, we performed Hb staining with o-dianisidine in tail fin amputated larvae during inflammation and resolution. We found accumulation of o-dianisidine^+^ dots at the injury site during inflammation (6hpa), which decreased sharply at resolution (24hpa, Supplementary Fig. S2D-E). Since o-dianisidine is a form of peroxidase stain that could also detect myeloperoxidase activity and consequently label neutrophils^19^, we perform tail fin amputations in the erythroid cell reporter *Tg(gata1a:DsRed)* to directly visualize RBCs *in vivo*. We found accumulation of *gata1a*^+^ cells at the injury which peaked during inflammation (6hpa), and decreased at resolution (24hpa, Fig. 1G-H), matching the kinetic observed with o-dianisidine staining. Altogether, these results indicate that RBCs accumulate at the injury site during the inflammatory phase after tail fin amputation in zebrafish larvae.

### Expression of heme oxygenase 1 is detected at the injury site of amputated larvae

**Fig. 2:**
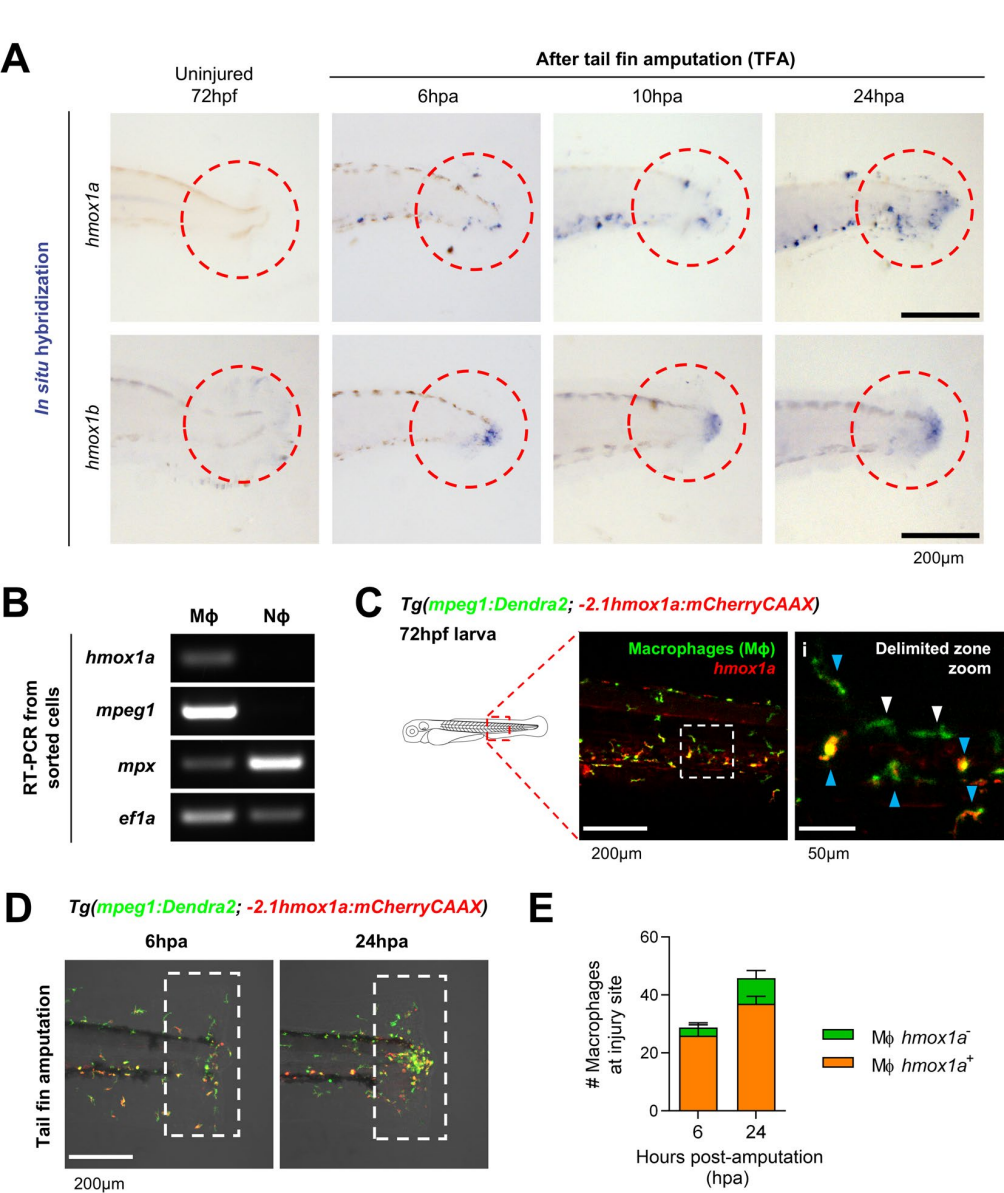
Expression of Hmox1 paralogs at the injury site of tail fin amputated zebrafish larvae. (**A**) *In situ* hybridization showing mRNA expression of the zebrafish paralogs *hmxo1a* and *hmox1b* at different timepoints following tail fin amputation. Representative images from 2 independent experiments are shown. Scale bar = 200μm. (**B**) Expression of *hmox1a* by RT-PCR from neutrophils (ΝΦ) and macrophages (ΜΦ) sorted from *Tg(mpx:GFP; mpeg1:Gal4FF; UAS:nfsB-mCherry)* larvae at 72hpf. Specific markers for neutrophils (*mpx*), macrophages (*mpeg1.1*), and housekeeping control (*ef1a*) are also shown. A representative result from 3 independent experiments is shown. (**C**) Confocal images from the tail of the double transgenic reporter *Tg(mpeg1:Dendra2; -2.1hmox1a:mCherryCAAX)*. A zoom of the CHT is shown in i. Blue arrowheads indicate macrophages positive for *hmox1a* (ΜΦ *hmox1a*^*+*^), whereas white arrowheads indicate macrophages negative for *hmox1a* (ΜΦ *hmox1a*^*-*^). Scale bar = 200μm for full picture (left) and 50μm for zoomed region (right). (**D**) Representative images showing recruitment of *hmox1a*^*+*^ macrophages to the injury site following tail fin amputation. Scale bar = 200μm. (**E**) Quantification of *hmox1a*^*+*^ macrophages recruited to the injury site following amputation (mean ± SD; n = 10 larvae per timepoint).

The decrease of RBCs at the injury site during resolution led us to hypothesize that an active mechanism oversees the removal of Hb from the injury site after tissue damage. The clearance of RBCs is linked with the degradation of Hb, which involves the breakdown of globin proteins into amino acids, and the catalysis of the heme ring by the Heme oxygenase 1 (HMOX1) enzyme into carbon monoxide (CO), iron (Fe), and biliverdin^20^. We therefore analyzed the expression of zebrafish Hmox1-coding genes at the injury site after damage. Through whole-mount *in situ* hybridization (WISH), we found enriched expression of both *hmox1a* and *hmox1b* paralogs at the injury site of amputated zebrafish larvae respect to non-amputated controls, in a time frame that coincided with the reduction of RBCs from the injury site from 6hpa to 24hpa (Fig. 2A). Interestingly, we found different expression patterns for the Hmox1 paralogs within the injury site: while the *hmox1a* signal was discrete and seemed restricted to individual cells, *hmox1b* expression was broadly expressed in the edge of the injured tissue (Fig. 2A). These results suggest that *hmox1b* expression is locally induced in the injured tissue, whereas *hmox1a* expression takes place in specific cells supporting the response to injury. As it has been previously described that *hmox1a* is the functional ortholog of the human *HMOX1* gene and that *hmox1b* is likely a pseudogene^21^, we decided to focus on the characterization of *hmox1a* at the injury site.

### Macrophages are the source of hmox1a at the injury site of amputated larvae

**Fig. 3:**
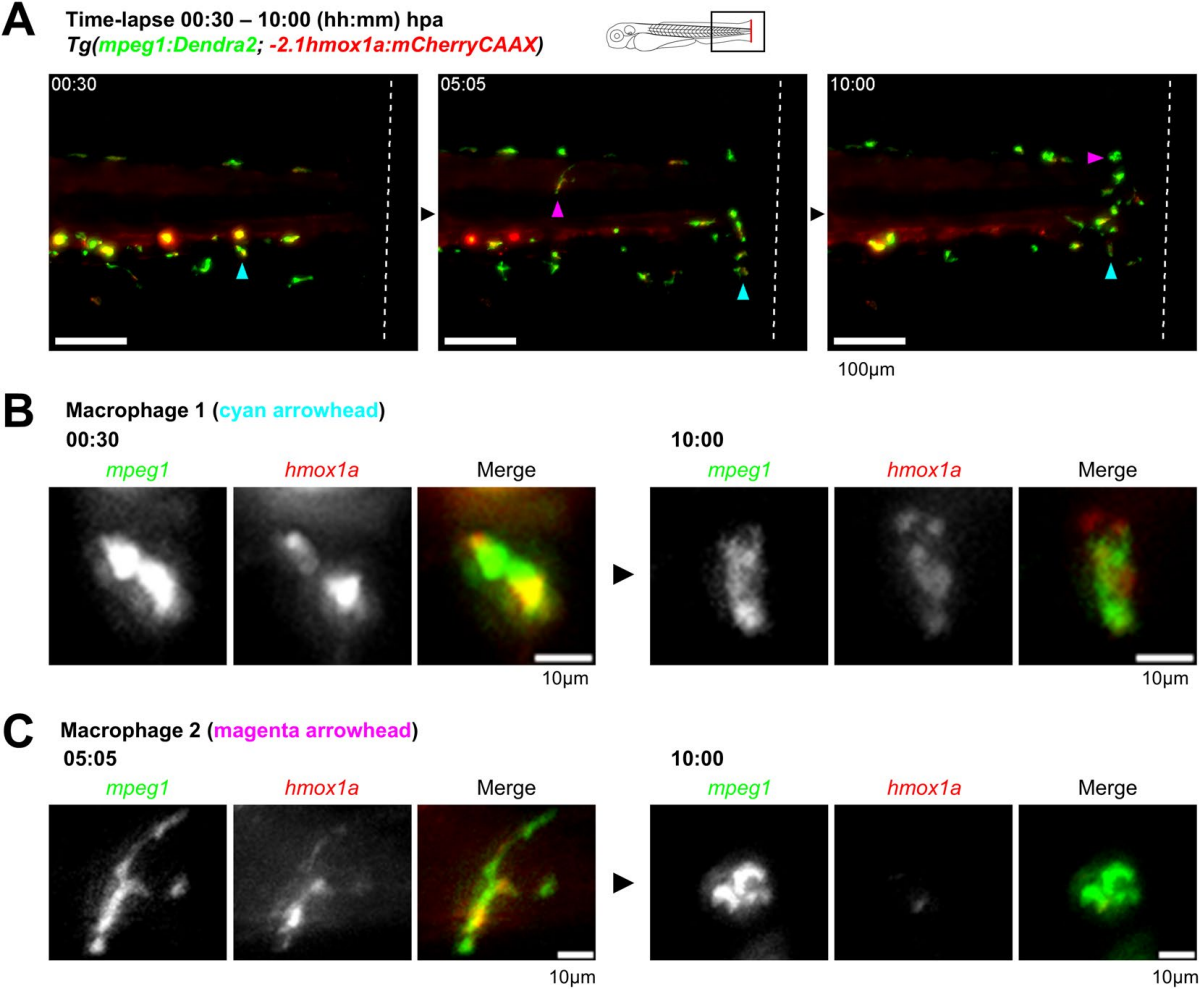
Migration of *hmox1a*^*+*^ macrophages to the injury site. (**A**) Time-lapse imaging of tail fin-amputated *Tg(mpeg1:Dendra2; -2.1hmox1a:mCherryCAAX)* larva from 30 minutes until 10 hours post-amputation (hpa), acquired at 5-minute intervals. Two individual macrophages, indicated by cyan and magenta arrowheads, are tracked over time. Time is shown as hh:mm. Scale bar = 100μm. (**B**) Macrophage 1 (cyan arrowhead from panel A) maintains *hmox1a* expression throughout the entire imaging period. (**C**) In contrast, macrophage 2 (magenta arrowhead from panel A) loses *hmox1a* expression toward the end of the imaging period. Scale bar = 10μm in B and C.

It has been previously reported that *hmox1a* is expressed in hematopoietic tissues of larvae^22,23^, and that is required for the proper function of macrophages^14^, which led us to hypothesize that macrophages are the source of *hmox1a* at the injury site of amputated larvae. By performing RT-PCR from FACS-sorted neutrophils and macrophages from the triple transgenic *Tg(mpx:GFP; mpeg1:Gal4; UAS:NTR-mCherry)* at 72hpf, we observed that macrophages, but not neutrophils, expressed *hmox1a* in homeostatic conditions (Fig. 2B, Supplementary Fig. S3). Single-cell analysis from the developing zebrafish atlas confirmed macrophages as the main source of *hmox1a* among immune and hematopoietic cells (Supplementary Fig. S4A). To further explore the kinetics of *hmox1a*^+^ cells *in vivo*, we generated two transgenic fluorescent reporters for *hmox1a* expression. For this, we cloned the 2.1kb genomic region immediately upstream of the initial ATG from the *hmox1a* in a Tol2-flanking plasmid containing the fluorescent proteins Dendra2 or membrane mCherry (mCherryCAAX) (Supplementary Fig. S4B, see methods). We injected these constructs into WT embryos, and after 2 generations we obtained the stable transgenic lines *Tg(-2.1hmox1a:Dendra2)* and *Tg(-2.1hmox1a:mCherryCAAX)*. We observed fluorescence expression of *hmox1a* in the posterior blood island (PBI) at 30hpf and caudal hematopoietic tissue (CHT) at 72hpf and 120hpf, in addition to expression in the lens and liver (Supplementary Fig. S3C), in line with observations made in a previously generated reporter for *hmox1a*^23^. Next, we crossed the generated *hmox1a* reporters with *Tg(lyz:DsRed*) and *Tg(mpeg1:Dendra2)* transgenic fish to analyze *hmox1a* expression in neutrophils and macrophages, respectively. We observed that macrophages were positive for *hmox1a* at 72hpf, while neutrophils were not (Fig. 2Ci, Supplementary Fig. S4D-F). Interestingly, we found variable expression levels of *hmox1a* in macrophages, as well as a group of macrophages that were negative for *hmox1a* (Fig. 2Cii). A more detailed analysis of the tail of 72hpf *Tg(mpeg1:Dendra2; -2.1hmox1a:mCherryCAAX)* larvae showed that 81.22% of tail macrophages expressed *hmox1a*, while the remaining 18.78% of tail macrophages were negative (Supplementary Fig. S4E). Next, we performed tail fin amputations in the double transgenic *Tg(mpeg1:Dendra2; -2.1hmox1a:mCherryCAAX)* at 72hpf to analyze *hmox1a* expression by macrophages at the injury site. We found that 90.28% of macrophages recruited to the injury site at 6hpa expressed *hmox1a*, but this proportion was reduced to 80.79% at 24hpa (Fig. 2D-E). Of note, the expression of *hmox1a* at the injury site was restricted to *mpeg1*^*+*^ macrophages in both timepoints, indicating that macrophages were the source of *hmox1a* expression at the injury site. Time-lapse imaging of amputated *Tg(mpeg1:Dendra2; -2.1hmox1a:mCherryCAAX)* from 30 minutes until 10hpa (Supplementary Video 1, Fig. 3A) revealed that macrophages expressed *hmox1a* before reaching the injury site (Fig. 3B-C). Interestingly, while most of recruited macrophages maintained *hmox1a* expression in the analyzed time window (Fig. 3B), we observed a macrophage that lost *hmox1a* expression while at the injury site (Fig. 3C), which could explain the increased frequency of *hmox1a*-negative macrophages observed at 24hpa.

### Inhibition of Hmox1 activity impairs RBC clearance and tail fin regeneration

**Fig. 4:**
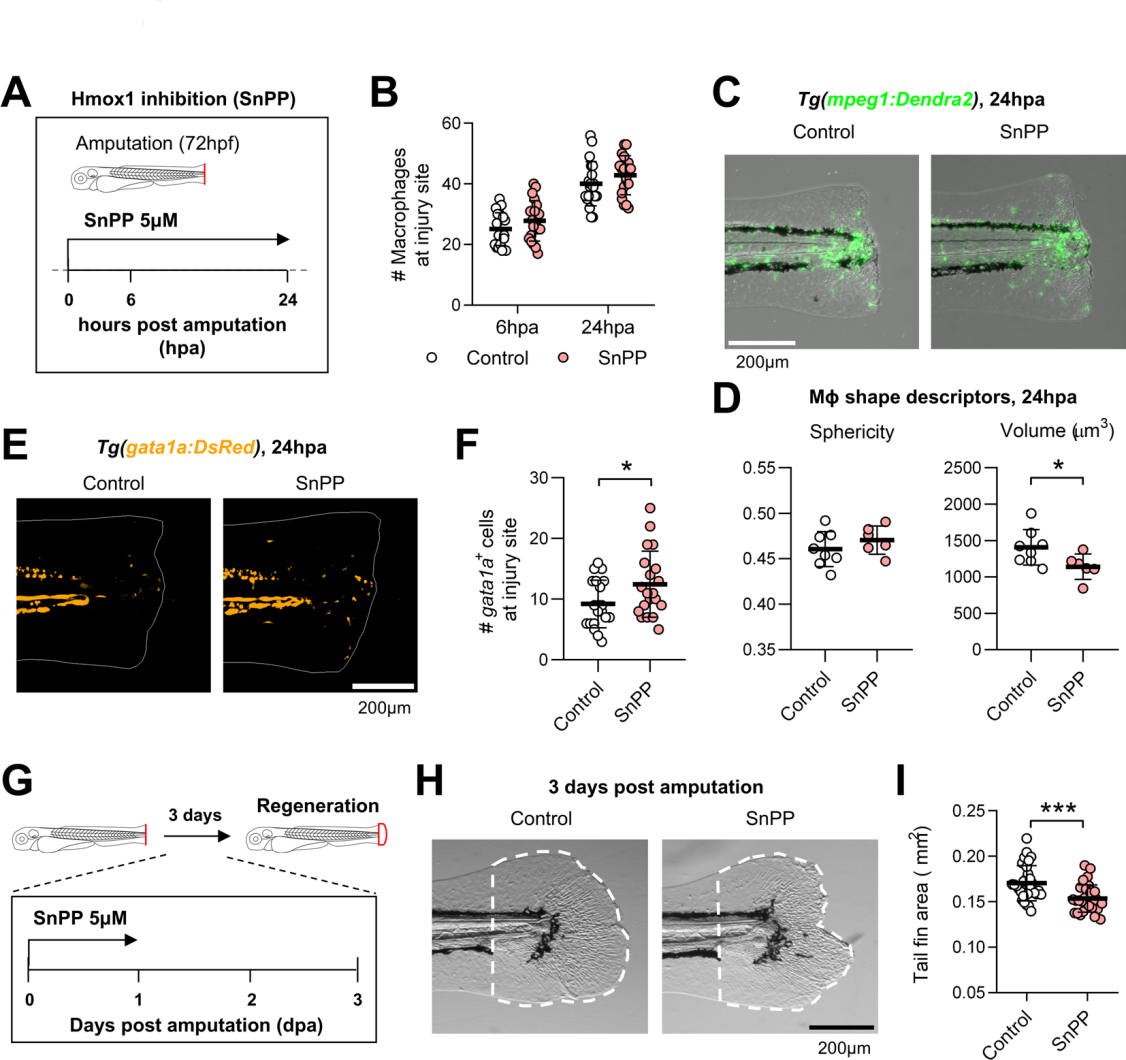
Inhibition of Hmox1 activity impairs red blood cell clearance from the injury site and affects tail fin regeneration. (**A**) Experimental design for quantification of amputated larvae treated with the Hmox1 inhibitor SnPP. (**B**) Quantification of macrophages recruited to the injury site in SnPP-treated amputated larvae (1 dot = 1 larva, 20 larvae per condition/timepoint). (**C**) Representative images of SnPP-treated *Tg(mpeg1:Dendra2)* amputated larvae at 24 hours post-amputation (hpa). Scale bar = 200μm. (**D**) Shape descriptors (sphericity and volume) of *mpeg1*^*+*^ macrophages recruited to the injury site in SnPP-treated larvae at 24hpa. Average values of recruited macrophages per larva are plotted (1 dot = 1 larva, 6-8 larvae per condition). (**E**) Representative fluorescence images of control and SnPP-treated *Tg(gata1a:DsRed)* larvae at 24hpa. Scale bar = 200μm. (**F**) Quantification of *gata1a*^+^ cells at the injury site of SnPP-treated larvae at 24hpa (1 dot = 1 larva, 20 larvae per condition). (**G**) Experimental design for tail fin regeneration analyses performed in SnPP-treated larvae. (**H**) Representative tail fins of amputated control and SnPP-treated larvae at 3 days post-amputation (3dpa). Scale bar = 200μm. (**I**) Quantification of the tail fin area of control and SnPP-treated larvae at 3dpa (1 dot = 1 larva, 30 larvae per condition).

To determine the functional consequences of Hmox1 activity after tail fin amputation, we took advantage of the Hmox1 inhibitor Tin protoporphyrin XI (SnPP), previously tested in zebrafish^14,24^, and we treated larvae for 24 hours immediately after amputation (Fig. 4A). Since a previous report suggested that macrophage recruitment to injury was impaired after Hmox1 activity inhibition^14^, we analyzed macrophage recruitment in our experimental settings. Although we did not observe differences in the number (Fig. 4B-C) nor their sphericity (Fig. 4D, left), we observed that recruited macrophages in SnPP-treated larvae had a smaller average volume compared to control larvae (Fig. 4D, right), suggesting that SnPP may affect macrophage physiology. Next, we analyzed the clearance of RBCs from the injury site after tail fin after amputation in SnPP-treated larvae. Inhibition of Hmox1 activity by SnPP led to an increased number of *gata1a*^*+*^ cells and o-dianisidine^+^ dots at 24hpa (Fig. 4E-F, Supplementary Fig. S5), indicating impaired RBC clearance from the injury site. We finally assessed whether inhibiting Hmox1 activity during the first 24h after amputation has long-term consequences and affects tail fin regeneration (Fig. 4G). We found that the regenerating tail fins of SnPP-treated larvae at 3 days post amputation (3dpa) were smaller in area when compared to their respective controls (Fig. 4H-I). Finally, to analyze whether the expression of *hmox1a* is sufficient to explain the observed phenotypes, we knocked down expression of *hmox1a* using a previously described morpholino^25^ (Supplementary Fig. S6A). We observed impaired RBC clearance during resolution (Supplementary Fig. S6B) and impaired tail fin regeneration (Supplementary Fig. S6C) in *hmox1a* morphants, phenocopying the effects observed following pharmacological inhibition of Hmox1 activity. Collectively, these findings indicate that Hmox1 promotes the clearance of RBCs from the injury site and the regeneration of the tail fin in larvae.

### Macrophage depletion increases RBCs at the injury site during the resolution of inflammation

**Fig. 5:**
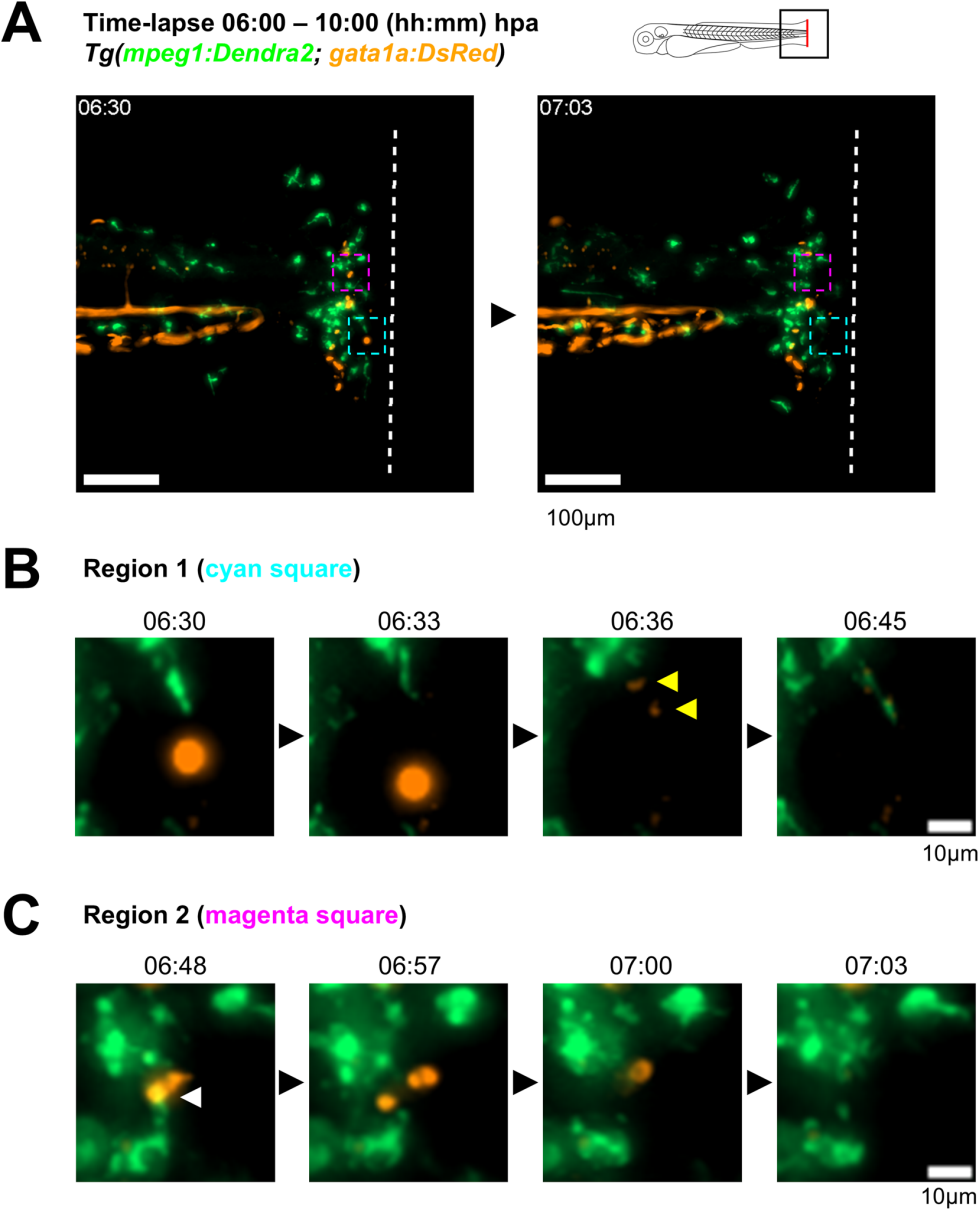
Interactions between red blood cells and macrophages at the injury site. (**A**) Time-lapse of tail fin-amputated *Tg(mpeg1:Dendra2; gata1a:DsRed)* larva from 6 until 10 hours post amputation (hpa), acquired at 3-minute intervals. Two regions of interest, indicated with dashed squares, were analyzed. Time is shown as hh:mm. Scale bar = 100μm. (**B**) In the first region (cyan dashed square in panel A), a *gata1a*^*+*^ red blood cell (RBC) disappeared between adjacent intervals (06:30 - 06:33), leaving small fluorescent fragments (yellow arrowheads at 06:36) that are engulfed by a neighbor macrophage at a later timepoint (06:45). (**C**) In the second region (magenta dashed squared in panel A), a *gata1a*^*+*^ RBC was in direct contact with a neighbor macrophage (white arrowhead at 06:48) before its fragmentation (06:57) and subsequent disappearance from the injury site (07:00 - 07:03). Scale bar = 10μm in B and C.

The importance of macrophages in the clearance of damage and pro-inflammatory cues, and our observations indicating that macrophages were the source of *hmox1a* at the injury site, led us to hypothesize that macrophages contribute to the clearance of RBCs from the from the injury site. Through time-lapse imaging of amputated *Tg(mpeg1:Dendra2; gata1a:DsRed)* reporter larvae from 6hpa to 10hpa (Supplementary Video 2, Fig. 5A), we analyzed interactions between macrophages and RBCs at the injury site. We observed that RBCs suddenly disappeared from the injury site, in some cases leaving behind small fluorescent particles in the surrounding tissue (Fig. 5B) or fragmenting into two parts before disappearing (Fig. 5C), consistent with localized hemolysis. Although we did not observe direct phagocytosis of RBCs, we found that macrophages were in close interaction with RBCs throughout the analyzed period and engulfed the debris generated following RBC disappearance (Fig. 5B). Furthermore, we observed macrophages interacting with RBCs immediately before their disappearance (Fig. 5C) suggesting that macrophages may facilitate RBC clearance through mechanisms independent of phagocytosis.

**Fig. 6:**
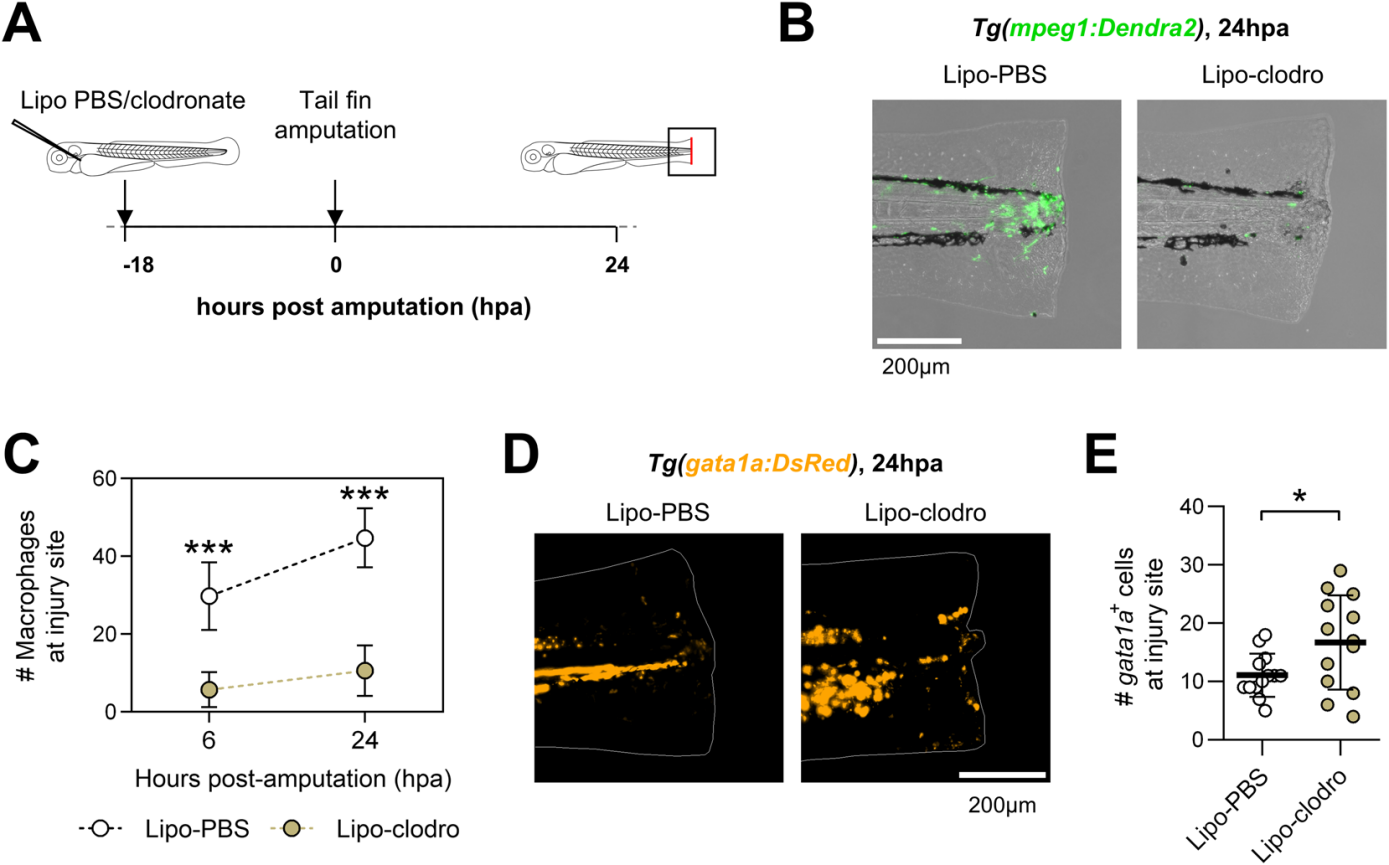
Macrophage depletion impairs red blood cell clearance from the injury site after tail fin amputation in larval zebrafish. (**A**) Experimental design for the depletion of macrophages using clodronate-loaded liposomes (Lipo-clodro). (**B**) Recruitment of macrophages in control (Lipo-PBS) and macrophage-depleted (Lipo-clodro) zebrafish at 24hpa. Representative images of the studied conditions are shown. Scale bar = 100μm. (**C**) Quantification of macrophages recruited to the injury site (mean ± SD; n = 25-26 larvae per condition/timepoint). (**D**) Representative fluorescent images of amputated Lipo-PBS- and Lipo-clodro-injected *Tg(gata1a:DsRed)* larvae at 24hpa. Scale bar = 200μm. (**E**) Quantification of *gata1a*^+^ cells at the injury site at 24hpa (1 dot = 1 larva, 13 larvae per condition).Unpaired t-tests were performed in C and E at specified timepoints. *p<0.05; ***p<0.001.

To functionally test the contribution of macrophages to RBC clearance, we depleted macrophages by injecting 2nL of 1:10 (0.5mg/mL) Lipo-clodronate and Lipo-PBS in the bloodstream of 54hpf zebrafish embryos (Fig. 6A), which we previously showed to deplete ~60% of macrophages in the tail and impair tail fin regeneration after amputation at 72hpf^10^. As expected, the recruitment of macrophages in Lipo-clodronate-injected larvae was markedly reduced at 6hpa and 24hpa compared to Lipo-PBS controls (Fig. 6B-C). Importantly, macrophage depletion resulted in an increased number of *gata1a*^+^ cells and o‑dianisidine^+^ dots at the injury site at 24hpa (Fig. 6D- E**, **Supplementary Fig. S7), phenocopying the effects observed upon inhibition of Hmox1. In conclusion, our findings support macrophages as active regulators of RBC clearance from the injury site following tail fin amputation in zebrafish larvae.

## Discussion

The availability of transgenic reporter lines and their optical transparency have made zebrafish larvae an ideal model to explore innate immune responses *in vivo*. While the focus has been directed toward neutrophils and macrophages, the potential involvement of additional hematopoietic-derived mediators during tissue injury has remained unexplored. Using an unbiased proteomic approach, we identified the accumulation of red blood cells (RBCs) at the injury site during inflammation, which subsequently cleared during resolution. In parallel, we observed the expression of the Hmox1 coding genes *hmox1a* and *hmox1b* at the injury site, and we identified macrophages as the source of injury-associated *hmox1a* expression after tail fin amputation. Pharmacological inhibition of Hmox1 activity with SnPP, morpholino-mediated knockdown of *hmox1a*, and the depletion of macrophages, led to increased RBC numbers at the injury site, supporting the role of *hmox1a*^*+*^ macrophages in RBCs clearance. Additionally, we found that the inhibition of Hmox1 activity and *hmox1a* expression led to impaired tail fin regeneration. Altogether, our findings indicate that Hmox1 is an important mediator of macrophage-dependent clearance of RBCs from the injury site and promotes tissue regeneration.

Our results show that zebrafish RBCs accumulate at the injury site after tail fin amputation. However, our reliance on o-dianisidine staining and *gata1a* reporter expression to detect RBCs in zebrafish tissues presents inherent limitations. Since o-dianisidine detects hemoglobin (Hb) based on peroxidase activity^26^, neutrophils can also be detected with this method, generating false-positive signals. Moreover, the *gata1a* fluorescent reporter is not fully specific to RBCs and also labels other erythroid-lineage cells, such as erythroid progenitors and thrombocytes. Consequently, both approaches are subject to unspecific background signals and can add noise to the collected results. Despite this, the *gata1a* fluorescent reporter yielded comparable results to o-dianisidine staining and supports the hypothesis that RBCs, rather than neutrophils or other erythroid-lineage cells, accumulate in injured tissues. While the primary function of RBCs is to provide oxygen to the injured tissue, oxygen exchange in zebrafish larvae occurs independently of RBCs^27,28^, which raises the question of what the role of RBCs is in this context. A previous study showed that nucleated RBCs from birds, amphibians, and fish, can reorganize their actin cytoskeleton during migration in a manner similar to leukocytes^29^. Additionally, fish RBCs were shown express genes associated with chemokine signaling and leukocyte transendothelial migration^30^, suggesting that they may exhibit chemotactic activity and respond to inflammatory cues. However, our study does not address the mechanisms by which zebrafish RBCs migrate to injured tissues or the signals that promote their extravascular migration, thereby opening new avenues for future research. It is known that RBCs are highly susceptible to hemolysis in extravascular spaces^31^, and the Hb released after hemolysis to the extracellular space may act as a DAMP capable of inducing oxidative stress, cytotoxicity, and amplifying inflammation through activation of toll-like receptor 4 (TLR4) signaling pathways^32^. Thus, while RBCs accumulation could be relevant to the initial steps of inflammation and likely protect the injured tissue from external pathogens^33^, the release of Hb in extravascular tissues poses a significant risk of cytotoxicity and exacerbation of inflammation, and therefore must be quickly cleared to avoid chronic inflammation. The temporal dynamics of RBCs observed following injury suggest that active RBC clearance is part of the resolution of inflammation, a required step for tissue regeneration. However, further studies are required to specifically address the role of zebrafish RBCs on tail fin regeneration.

We observed the expression of the Hmox1-coding genes *hmox1a* and *hmox1b* in the injury site, which correlated with the peak and reduction of RBCs in the injury site. However, interpretative caveats must be considered regarding the expression patterns of *hmox1a* and *hmox1b* revealed by *in situ* hybridization. As no control probes were used in the experiment, this raises the possibility that non-specific accumulation occurs in the wound edge, particularly for *hmox1b*. In the case of *hmox1a*, the generated transgenic fluorescent reporter validated the *in situ* hybridization patterns observed following injury and confirms that *hmox1a* is actively expressed by cells in the injury site. Although our findings led us to propose that Hmox1 is a critical mediator of RBC clearance following injury, it is also possible that the anti-oxidant function of Hmox1 activity directly regulates the inflammatory responses in the injury site, as previous studies have suggested^34,35^, and further analysis of pro-inflammatory cytokine expression and reactive oxygen species (ROS) production are required to better understand the relationship between Hmox1 activity and inflammatory responses. Since a previous study showed that *hmox1a* is the functional ortholog of the human *HMOX1* gene ^21^, we focused our attention on characterizing the source of *hmox1a* expression following injury. However, we cannot rule out potential regulatory functions of *hmox1b* in the injury site, such as small interfering RNA, competitive endogenous RNA, or antisense transcripts^36^, which could ultimately modulate the tissue response to injury at a transcriptional level. Our results showed that macrophages are an important, but not exclusive, source of *hmox1a* in zebrafish during steady state conditions. However, macrophages were the sole source of *hmox1a* in the injury site after tail fin amputation, with most of the macrophages being *hmox1a*^*+*^. The use of a novel *hmox1a* fluorescent reporter line in our study provided an unprecedented view of *hmox1a* expression dynamics *in vivo*. A previous study described a fluorescent reporter for *hmox1a*^23^, and the expression pattern described in the rostral blood island, liver, and retina matched our observations. However, they attributed *hmox1a* expression to erythroid lineages and not to macrophages, as we showed in this study. Our observations are supported by previous evidence showing functional defects of macrophages in *hmox1a* mutants^14^ and single-cell transcriptomic analysis showing expression of *hmox1a* in macrophages at the CHT^37^. Thus, our reporter enabled real-time visualization and spatial resolution of *hmox1a*-expressing macrophages in the context of tissue injury and offers a valuable advantage for future studies aiming to dissect the precise temporal and cellular dynamics of heme metabolism and immune regulation during homeostasis, tissue injury, and repair.

As our findings show that 1) macrophages are the sole source of *hmox1a* expression at the injury site, 2) both inhibition of Hmox1 activity and *hmox1a* expression lead to an impaired clearance of RBCs from the injury site, and 3) depletion of macrophages similarly results in impaired RBC clearance, we propose that *hmox1a*-expressing macrophages are critical mediators of RBC clearance after tissue injury. Crucially, both SnPP-treated larvae and *hmox1a* morphants exhibited impaired tail fin regeneration following amputation, consistent with an effector role of Hmox1a in macrophages promoting tissue regeneration. This interpretation is further supported by cumulative evidence from murine models of injury showing that *HMOX1* expression drives macrophage polarization towards anti-inflammatory profiles^12^ and endows them with pro-regenerative functions^13^. Although we observed that macrophages expressed *hmox1a* prior to their recruitment to the injury site, the exact transcriptional profile of *hmox1a*^*+*^ macrophages in homeostasis, and whether it changes at the injury site following interaction with RBCs remains to be elucidated. Our findings also suggest that macrophages promote RBC clearance independent of phagocytosis, potentially by facilitating hemolysis. However, a more comprehensive characterization is required to elucidate the cellular and molecular basis of this observation. Moreover, as our time-lapse analysis was restricted to a very narrow time window, we cannot exclude the possibility that direct phagocytosis of RBCs occurs at later stages after injury. Future studies combining high-resolution imaging of macrophage-RBC interactions with single-cell transcriptomics and ligand-receptor interaction analyses will be critical to define the cellular choreography underpinning clearance of RBCs, resolution of inflammation, and tissue regeneration.

Although this work was conducted in zebrafish larvae, our findings could be relevant for human wound healing. Previous studies in mammals have demonstrated that induction of HMOX1 accelerates wound healing, including in models of diabetic ulcers, supporting its therapeutic potential in chronic, non-healing human wounds^38,39^. Our findings show that the protective role of HMOX1 is conserved in zebrafish and support the use of this model to further understand the molecular mechanisms underlying Hmox1 activity in wounds. Moreover, our work opens a new avenue for investigating the role of RBCs and Hb/heme during tissue injury. While the function of RBCs has traditionally been limited to oxygen supply during injury, recent evidence from humans, birds, and fish suggest they can exert active immunological functions, such as chemokine regulation, nucleic acid sensing, and pathogen binding^33^. Notably, pathologies affecting RBC integrity or turnover, such as anemia and hemoglobinopathies, are often associated with impaired wound healing and increased risk of wound infection^40^. These observations raise the possibility that defects in RBC integrity or turnover may directly contribute to chronic or impaired wound healing. Collectively, these findings support further investigation into the oxygen-independent immunological roles of RBCs in tissue repair.

Despite the insights acquired from our study, additional limitations should be acknowledged. First, although we inferred the inflammatory and resolution phases following injury based on immune cell dynamics — specifically neutrophil presence — we relied on using specific timepoints associated with these phases (6hpa and 24hpa), omitting a comprehensive kinetic evaluation at multiple timepoints following injury. Additionally, assessing the expression of common pro- and anti-inflammatory cytokines (such as *tnfa*, *il1b*, and *il10*) would further aid in delineating the inflammatory and resolution phases following injury. A better-defined characterization of the inflammatory milieu would help to further clarify timing and polarization of inflammatory responses and validate the proposed role of Hmox1a in inflammation resolution. Second, the expression of the *hmox1a* fluorescent reporters to track *hmox1a*-expressing cells should be carefully analyzed, as fluorescence proteins could persist in a cell even after *hmox1a* transcription has ceased. As such, the reporter may reflect prior, but not necessarily current, transcriptional activity. Conversely, during early stages of *hmox1a* induction, the reporter may not yet have accumulated to a detectable threshold—leading to potential underrepresentation of actively transcribing cells. This temporal disconnect between transcriptional dynamics and reporter signal could obscure transient or low-level expression events and must be considered in the interpretation of the data. Finally, our work does not provide evidence to establish a causal link between RBC clearance and tail fin regeneration. It therefore remains possible that Hmox1-dependent mechanisms other than Hb/heme degradation contribute to the observed phenotypes. Further studies that directly manipulate RBC or Hb abundance in zebrafish are required to determine their exact role during tail fin regeneration.

In conclusion, our findings emphasize *hmox1a* as a macrophage-associated effector essential for the resolution of inflammation after injury and the promotion of tissue regeneration in zebrafish. Additionally, it uncovers RBCs as potential players of wound inflammatory responses, which may have implications in the study of immune responses of patients suffering from RBC disorders.

## Methods

### Fish husbandry and lines

Zebrafish (*Danio rerio*, strains TAB5, AB, and Tübingen) were reared and kept, according to standard procedures, in the zebrafish facilities at the Facultad de Ciencias of Universidad de Chile, the FishCore at Monash University, and the Karolinska Institutet Zebrafish Core Facility. Adult zebrafish, used to obtain embryos for experiments, were maintained at 28 °C in recirculating, filtered water systems (Techniplast, Italy; Aquaneering, USA), on a regimen of 14 hours of light and 10 hours of darkness. Established zebrafish lines used in this work were: *Tg(fli1:EGFP)*^*y1*^^41^; *Tg(mpeg1:Gal4FF)*^*gl25*^^42^ combined with *Tg(UAS-E1b:NfsB-mCherry)*^*c264*^^43^, referred to as *Tg(mpeg1:Gal4; UAS:NTR-mCherry)*; *TgBAC(mpx:GFP)*^*i114*^, referred to as *Tg(mpx:GFP)*^44^; *Tg(mpx:mCherry)*^*udc1*^^45^; *Tg(lyz:DsRed2)*^*nz50*^, referred to as *Tg(lyz.DsRed)*^46^; *Tg(gata1a:DsRed)*^*sd2*^^47^. Zebrafish transgenic lines that were reestablished by injecting the constructs were: *Tg(mpeg1:EGFP)*^42^ and *Tg(mpeg1:Dendra2)* (gift from Dr. Anna Huttenlocher, Addgene plasmid #51462)^48^. Zebrafish embryos were collected by natural spawning of adults and were kept at 28°C in E3 water (NaCl 5mM, KCl 0.17mM, CaCl_2_ 0.33mM, MgSO_4_ 0.33mM). Embryos were checked daily, and specimens showing any signs of malformations or developmental delays were discarded and not used for experiments. All procedures were performed in accordance with the “Guidelines for the Use of Fishes in Research Use” of the American Fisheries Society (www.fisheries.org) and complied with local regulations. Husbandry, breeding of zebrafish stocks for the collection of embryos, and procedures performed in zebrafish older than 5 days post fertilization were approved by the Animal Ethics Committee of the Universidad de Chile (approval: 2015-04-20). Husbandry and breeding of zebrafish stocks for the collection of embryos were approved by the Monash University Animal Ethics Committee (protocol MAS-2010-18) and the Stockholm Ethical Committee from the Swedish Board of Agriculture (diary number 15591-2023). This study is reported in accordance with the ARRIVE guidelines.

### Tail fin amputation and in vivo imaging

At 72 hours post fertilization (hpf), larvae were anesthetized with 0.016% MS-222, and the tail fin was amputated with a scalpel, using the caudal vein loop and the posterior section of the ventral pigmentation gap in the tail as references^10^. The amputation did not cause damage to either the caudal vein loop or to blood vessels near the injury site. Immediately after amputation, larvae were rinsed and incubated in E3 medium at 28°C. Quantifications, imaging of recruited immune cells to the injury site (up to ∼200μm from the amputation site), and imaging of the regenerating tail fin were performed using an Olympus MVX10 and Leica M165 FC fluorescence stereomicroscopes, Olympus IX81 epifluorescence microscope, and Zeiss Axiovert 200 widefield microscope. Quantifications from images were performed using the “Cell Counter” plugin in Fiji/ImageJ software (NIH, USA). Time-lapse imaging of amputated fluorescent transgenic reporter lines was performed as previously described^10^. Briefly, larvae were mounted in 0.8% low melting point agarose solution with 0.01% MS-222. Time-lapses were performed using Olympus IX81 and Zeiss Cell Observer widefield microscopes. After imaging, larvae were euthanized by incubation with an overdose of MS-222 (ranging between 0.03% - 0.1%) for 30 minutes, followed by freezing of individuals.

### Tissue collection and proteomic analyses

At 6 and 24 hours post amputation (hpa), larvae were euthanized with an overdose of MS-222, and tail tissue pieces were collected by performing a second transection at approximately 200-300µm from the injury site, using the narrowest region of the larval fin fold as a reference. Single replicates containing tail tissue pieces from 200 larvae/condition/timepoint were collected in 1.5mL Eppendorf tubes, which were spun before removing all the media, snap-frozen in dry ice-cooled methanol, and stored at -80°C. At each time point, tissues from non-amputated controls were collected for comparisons. Samples were subsequently submitted to a global proteomic profiling of 10.000 sequencing events, followed by sequence library searching (service provided by Bioproximity LLC, USA). Briefly, proteins from samples were extracted and sequenced by Shotgun proteomics followed by Tandem Mass Spectrometry (MS/MS) in a Q-Exactive HF-X Orbitrap mass spectrometer. Identified peptides, using OMSSA and X!Tandem algorithms, were aligned to the zebrafish proteome, and UniProt (Universal Protein Resource) identification codes and gene names were obtained. The identification of proteins exclusively present in the injury site at the studied timepoints was performed in 3 steps: 1) First, identified UniProt IDs were converted to Entrez ID using the DAVID Bioinformatic resource v6.8^16,49^, and redundant Entrez IDs were excluded; 2) common and sample-specific Entrez IDs were identified in samples from injured tissues (TFA) were compared to their respective time-matched non-injured controls using an online Venn diagram generator (https://bioinformatics.psb.ugent.be/webtools/Venn/), and injury-specific Entrez-ID were selected; and 3) common and timepoint-specific Entrez IDs (inflammation or resolution) were identified. Finally, functional annotation clustering of the proteins exclusively present in the injury site at the specific time point was performed using the DAVID Bioinformatic resource v6.8, in which functional annotations, gene ontology (GO), pathways (KEGG^50,51^, permission granted by Kanehisa Laboratories), and protein domains were considered. The classification was performed using the highest stringency settings.

### Hemoglobin staining in zebrafish larvae

Zebrafish larvae were stained with o-dianisidine (Sigma-Aldrich) before or after fixation with PFA 4%, based on previously described protocols^52,53^. Before imaging, larvae were depigmented with a solution of H_2_O_2_ 3% v/v and KOH 1% w/v in deionized water. Color images were acquired using Olympus MVX10 and Nikon SMZ25 stereomicroscopes, and the number of o-dianisidine^+^ dots at the injury site was quantified using the “Cell Counter” plugin in Fiji/ImageJ.

### Whole-mount in situ hybridization

For *in situ* hybridization experiments, larvae were incubated from 24hpf in E3 supplemented with phenyl thiourea (PTU), and amputations were performed using larvae collected before amputation (72hpf) and after 6h, 10h, and 24h post-amputation. Larvae were fixed in PFA 4%, dehydrated in methanol, and incubated for at least 1 night at -20°C. Whole-mount *in situ* hybridizations were performed as previously described^54^. For imaging, stained larvae were transferred to glycerol and imaged with an MVX10 stereoscope (Olympus). The pBSKII SK plasmid containing the antisense probe against *hmox1a* was kindly provided by Dr. Makoto Kobayashi^55^, whereas the antisense probe for *hmox1b* was generated by cloning a portion of the *hmox1b* gene into a pBSK vector, as previously described^56^. Primers used for the cloning of the *hmox1b* probe are found in Supplementary Table S4. No control probes were used in these experiments.

### Analysis of single-cell RNAseq databases

For expression analysis, we took advantage of the single-cell atlas of the developing zebrafish embryo^18^. The Seurat file containing the clustered single cells was downloaded from https://www.adammillerlab.com/resources. Hematopoietic cells, including neutrophils (cluster 150), macrophages (clusters 71, 184, and 212), thymocytes (cluster 59), RBCs (clusters 6, 38, 48, 63, 85, and 157), and RBC progenitors (cluster 153) were selected for further analysis. Gene expression analyses were performed using Seurat v4.0 in RStudio.

### Sorting of immune cells from larvae and RT-PCR

At 72hpf, transgenic reporter larvae *Tg(mpx:GFP; mpeg1:Gal4FF; UAS:NTR-mCherry)* were disaggregated to single-cell suspensions as previously reported^57^. Sorting of neutrophils (GFP^+^) and macrophages (mCherry^+^) was performed using an Influx2 Cell Sorter (BD Biosciences) at the Monash University FlowCore unit. Total RNA was isolated from sorted cells using the RNeasy mini kit (Qiagen), and RT-PCR analyses were performed using the SuperScript III one-step RT-PCR system (Invitrogen). Primers used for RT-PCR are shown in Supplementary Table S4.

### Generation of the hmox1a transgenic reporter line

The 2,297bp genomic sequence containing the upstream and first 45 nucleotides of the *hmox1a* coding sequence (Gene ID: 791518) was amplified and cloned into a pCRII vector. A second PCR was performed on the previously cloned sequence to amplify a 2,151bp sequence containing the upstream and first nine nucleotides of the *hmox1a* coding sequence, flanked by restriction enzyme sites for XhoI and Cfr9I. Primers used for PCR amplifications are shown in Supplementary Table S4. All the PCRs were performed using the Platinum *Taq* DNA polymerase high fidelity (Invitrogen). The plasmid Tol2_*mpeg1:Dendra2* (Addgene, plasmid #51462)^48^, containing the Dendra2 fluorescent protein flanked by Tol2 elements, was co-digested by XhoI/Cfr9I (Thermo Fisher) to remove the original *mpeg1* promoter and ligate the cloned 2.1kb *hmox1a* promoter, thus generating the Tol2-based *-2.1hmox1a:Dendra2* construct. The cloning strategy used left the first 3 amino acids of the *hmox1a* coding sequence in-frame with the Dendra2 coding sequence. Cloning was verified by restriction enzyme analysis and Sanger sequencing (Macrogen, South Korea). For the generation of the Tol2-based *-2.1hmox1a:mCherryCAAX* construct, the Dendra2 coding sequence of the generated vector was excised using Cfr9I and MunI restriction enzymes and replaced by a PCR product containing the mCherryCAAX coding sequence flanked by Cfr9I and MunI. For the generation of the *hmox1a* transgenic lines, 1-2nL of injection solution containing the generated plasmids (25ng/µL) and the Tol2 transposase mRNA (35ng/µL) was injected into 1-cell stage wild-type zebrafish zygotes. Positive larvae for Dendra2 or mCherryCAAX (F0 generation) were raised to adults, and founders were identified after outcrossing F0 adults with non-fluorescent wild-type fish. Larvae used for experiments were from the F2 generation onwards.

### Chemical treatments

The Hmox1 inhibitor Tin protoporphyrin XI (SnPP, Cat. #0747 from Tocris and #16375 from Cayman Chemical) was resuspended in DMSO to a working stock of 5mM. Immediately after tail fin amputations, larvae were randomly separated into two groups. The first group (treatment) was treated with SnPP at a concentration of 5µM (1:1000 from working stock). The second group of larvae (control) was treated with E3 supplemented with DMSO 0.1%. Treatments were performed for 24 hours, after which larvae were rinsed three times with E3.

### Morpholino (MO) experiments

For knockdown experiments, a previously described morpholino targeting the translation start of the *hmox1a* gene^25^ was used. Briefly, 1-2nL of a solution containing 0.8 µg/µL of *hmox1a* MO (5’-AGTCCATCTTTGTGCTGTAGATGTC-3’, Gene Tools LLC, USA) and 0.05% of phenol red (Sigma-Aldrich) was injected into 1- to 2-cell stage zygotes. Injections of equal doses of Standard Control Oligo (5’-CCTCTTACCTCAGTTACAATTTATA-3’, GeneTools LLC) were performed in control morphants. Morpholino-injected embryos showing no signs of edema or malformations were selected for experiments.

### Depletion of macrophages with clodronate liposomes

Clodronate-loaded liposomes (www.clodronateliposomes.com) were used to reduce the macrophage pool of zebrafish larvae. At 54hpf, zebrafish larvae were randomly separated into two groups. The first group of larvae was injected with 2nL of 0.5mg/mL clodronate-loaded liposomes (Lipo-clodronate, 1:10 from stock) into the bloodstream through the circulation valley. The second group of larvae was injected with an equivalent dilution of PBS-loaded liposomes (Lipo-PBS). Amputations were conducted 18 hours after Lipo-clodronate or Lipo-PBS injections.

### Statistical analysis

Statistical tests were performed using Prism 8.0 software (GraphPad, USA). Differences between samples were considered significant when the obtained p-value was lower than 0.05.

## Supplementary Information

Supplementary Information 1

Supplementary Information 2

Supplementary Information 3

Supplementary Information 4

Supplementary Information 5

Supplementary Video 1

Supplementary Video 2

## Data Availability

Shotgun proteomic raw files are publicly available in the MassIVE proteomic repository (accession number: MSV000092719). Additional data can be requested from the corresponding author on reasonable request.

## References

[1] Koh, T. J. & DiPietro, L. A. Inflammation and wound healing: the role of the macrophage. *Expert Rev. Mol. Med.* **13**, e23 (2011). 21740602 10.1017/S1462399411001943 PMC3596046

[2] Wilgus, T. A., Roy, S. & McDaniel, J. C. Neutrophils and wound repair: positive actions and negative reactions. *Adv. Wound Care* **2**, 379–388 (2013). 10.1089/wound.2012.0383 PMC3763227 24527354

[3] Wynn, T. A. & Vannella, K. M. Macrophages in tissue repair, regeneration, and fibrosis. *Immunity* **44**, 450–462 (2016). 26982353 10.1016/j.immuni.2016.02.015 PMC4794754

[4] Ellett, F. & Lieschke, G. J. Zebrafish as a model for vertebrate hematopoiesis. *Curr. Opin. Pharmacol.* **10**, 563–570 (2010). 20538521 10.1016/j.coph.2010.05.004

[5] van der Vaart, M., Spaink, H. P. & Meijer, A. H. Pathogen recognition and activation of the innate immune response in zebrafish. *Adv. Hematol* **2012**, 159807 (2012). 22811714 10.1155/2012/159807 PMC3395205

[6] Franza, M. et al. Zebrafish (*Danio rerio*) as a model system to investigate the role of the innate immune response in human infectious diseases. *Int. J. Mol. Sci.* **25**, 12008 (2024). 39596075 10.3390/ijms252212008 PMC11593600

[7] Li, L., Yan, B., Shi, Y.-Q., Zhang, W.-Q. & Wen, Z.-L. Live imaging reveals differing roles of macrophages and neutrophils during zebrafish tail fin regeneration. *J. Biol. Chem.* **287**, 25353–25360 (2012). 22573321 10.1074/jbc.M112.349126 PMC3408142

[8] Petrie, T. A., Strand, N. S., Yang, C.-T., Rabinowitz, J. S. & Moon, R. T. Macrophages modulate adult zebrafish tail fin regeneration. *Development* **141**, 2581–2591 (2014). 24961798 10.1242/dev.098459 PMC4067955

[9] Nguyen-Chi, M. et al. TNF signaling and macrophages govern fin regeneration in zebrafish larvae. *Cell Death Dis.* **8**, e2979 (2017). 28796253 10.1038/cddis.2017.374 PMC5596562

[10] Morales, R. A. & Allende, M. L. Peripheral macrophages promote tissue regeneration in zebrafish by fine-tuning the inflammatory response. *Front. Immunol.* **10**, 253 (2019). 30891030 10.3389/fimmu.2019.00253 PMC6413720

[11] Hasegawa, T. et al. Transient inflammatory response mediated by interleukin-1β is required for proper regeneration in zebrafish fin fold. *Elife* **6**, e22716 (2017). 28229859 10.7554/eLife.22716 PMC5360449

[12] Zhang, M. et al. Myeloid HO-1 modulates macrophage polarization and protects against ischemia-reperfusion injury. *JCI Insight* **3**, e120596 (2018). 30282830 10.1172/jci.insight.120596 PMC6237471

[13] Patsalos, A. et al. The BACH1-HMOX1 regulatory axis is indispensable for proper macrophage subtype specification and skeletal muscle regeneration. *J. Immunol.* **203**, 1532–1547 (2019). 31405954 10.4049/jimmunol.1900553 PMC6736746

[14] Luo, K. et al. Zebrafish heme oxygenase 1a is necessary for normal development and macrophage migration. *Zebrafish* **19**, 7–17 (2022). 35108124 10.1089/zeb.2021.0058

[15] Gray, C. et al. Simultaneous intravital imaging of macrophage and neutrophil behaviour during inflammation using a novel transgenic zebrafish. *Thromb. Haemost.* **105**, 811–819 (2011). 21225092 10.1160/TH10-08-0525

[16] Huang, D. W., Sherman, B. T. & Lempicki, R. A. Systematic and integrative analysis of large gene lists using DAVID bioinformatics resources. *Nat. Protoc.* **4**, 44–57 (2009). 19131956 10.1038/nprot.2008.211

[17] Pei, W. et al. Extracellular HSP60 triggers tissue regeneration and wound healing by regulating inflammation and cell proliferation. *npj Regen. Med.* **1**, 16013 (2016). 28936359 10.1038/npjregenmed.2016.13 PMC5605149

[18] Farnsworth, D. R., Saunders, L. M. & Miller, A. C. A single-cell transcriptome atlas for zebrafish development. *Dev. Biol.* **459**, 100–108 (2020). 31782996 10.1016/j.ydbio.2019.11.008 PMC7080588

[19] Xie, R. et al. Microparticles in red cell concentrates prime polymorphonuclear neutrophils and cause acute lung injury in a two-event mouse model. *Int. Immunopharmacol.* **55**, 98–104 (2018). 29241160 10.1016/j.intimp.2017.11.029

[20] Ryter, S. W., Alam, J. & Choi, A. M. K. Heme oxygenase-1/carbon monoxide: from basic science to therapeutic applications. *Physiol. Rev.* **86**, 583–650 (2006). 16601269 10.1152/physrev.00011.2005

[21] Luo, K., Stocker, R., Britton, W. J., Kikuchi, K. & Oehlers, S. H. Haem oxygenase limits *Mycobacterium marinum* infection-induced detrimental ferrostatin-sensitive cell death in zebrafish. *FEBS J.* **289**, 671–681 (2022). 34544203 10.1111/febs.16209

[22] Craven, S. E., French, D., Ye, W., de Sauvage, F. & Rosenthal, A. Loss of Hspa9b in zebrafish recapitulates the ineffective hematopoiesis of the myelodysplastic syndrome. *Blood* **105**, 3528–3534 (2005). 15650063 10.1182/blood-2004-03-1089

[23] Holowiecki, A., O’Shields, B. & Jenny, M. J. Spatiotemporal expression and transcriptional regulation of heme oxygenase and biliverdin reductase genes in zebrafish (*Danio rerio*) suggest novel roles during early developmental periods of heightened oxidative stress. *Comp. Biochem. Physiol. C Toxicol. Pharmacol.* **191**, 138–151 (2017). 27760386 10.1016/j.cbpc.2016.10.006 PMC5148680

[24] Li, Z.-X. et al. Xyloketal B exhibits its antioxidant activity through induction of HO-1 in vascular endothelial cells and zebrafish. *Mar. Drugs* **11**, 504–522 (2013). 23429283 10.3390/md11020504 PMC3640395

[25] Tzaneva, V. & Perry, S. F. Evidence for a role of heme oxygenase-1 in the control of cardiac function in zebrafish (*Danio rerio*) larvae exposed to hypoxia. *J. Exp. Biol.* **219**, 1563–1571 (2016). 26994186 10.1242/jeb.136853

[26] O’brien, B. R. A. Identification of haemoglobin by its catalase reaction with peroxide and o-Dianisidine. *Stain Technol.* **36**, 57–61 (1961).

[27] Pelster, B. & Burggren, W. W. Disruption of hemoglobin oxygen transport does not impact oxygen-dependent physiological processes in developing embryos of zebra fish (*Danio rerio*). *Circ. Res.* **79**, 358–362 (1996). 8756015 10.1161/01.res.79.2.358

[28] Rombough, P. & Drader, H. Hemoglobin enhances oxygen uptake in larval zebrafish (*Danio rerio*) but only under conditions of extreme hypoxia. *J. Exp. Biol.* **212**, 778–784 (2009). 19251992 10.1242/jeb.026575

[29] Chernyavskikh, S. D., Fedorova, M. Z., Van Thanh, V. & Quyet, D. H. Reorganization of actin cytoskeleton of nuclear erythrocytes and leukocytes in fish, frogs, and birds during migration. *Cell Tissue Biol.* **6**, 348–352 (2012). 22827038

[30] Shen, Y., Wang, D., Zhao, J. & Chen, X. Fish red blood cells express immune genes and responses. *Aquaculture Fish.* **3**, 14–21 (2018).

[31] Jeney, V., Eaton, J. W., Balla, G. & Balla, J. Natural history of the bruise: formation, elimination, and biological effects of oxidized hemoglobin. *Oxid. Med. Cell Longev.* **2013**, 703571 (2013). 23766858 10.1155/2013/703571 PMC3671564

[32] Dutra, F. F. & Bozza, M. T. Heme on innate immunity and inflammation. *Front. Pharmacol.* **5**, 115 (2014). 24904418 10.3389/fphar.2014.00115 PMC4035012

[33] Anderson, H. L., Brodsky, I. E. & Mangalmurti, N. S. The evolving erythrocyte: RBCs as modulators of innate immunity. *J. Immunol.* **201**, 1343–1351 (2018). 30127064 10.4049/jimmunol.1800565 PMC6108441

[34] Wagener, F. A. D. T. G. et al. Different faces of the heme-heme oxygenase system in inflammation. *Pharmacol. Rev.* **55**, 551–571 (2003). 12869663 10.1124/pr.55.3.5

[35] Willis, D., Moore, A. R., Frederick, R. & Willoughby, D. A. Heme oxygenase: a novel target for the modulation of the inflammatory response. *Nat. Med.* **2**, 87–90 (1996). 8564848 10.1038/nm0196-87

[36] Tasnim, M., Wahlquist, P. & Hill, J. T. Zebrafish: Unraveling genetic complexity through duplicated genes. *Dev. Genes Evol.* **234**, 99–116 (2024). 39079985 10.1007/s00427-024-00720-6 PMC11612004

[37] Wattrus, S. J. et al. Quality assurance of hematopoietic stem cells by macrophages determines stem cell clonality. *Science* **377**, 1413–1419 (2022). 36137040 10.1126/science.abo4837 PMC9524573

[38] Grochot-Przeczek, A. et al. Heme oxygenase-1 accelerates cutaneous wound healing in mice. *PLoS ONE* **4**, e5803 (2009). 19495412 10.1371/journal.pone.0005803 PMC2686151

[39] Leal, E. C. & Carvalho, E. Heme oxygenase-1 as therapeutic target for diabetic foot ulcers. *Int. J. Mol. Sci.* **23**, 12043 (2022). 36233341 10.3390/ijms231912043 PMC9569859

[40] Kavanagh, P. L., Fasipe, T. A. & Wun, T. Sickle cell disease: a review. *JAMA* **328**, 57–68 (2022). 35788790 10.1001/jama.2022.10233

[41] Lawson, N. D. & Weinstein, B. M. In vivo imaging of embryonic vascular development using transgenic zebrafish. *Dev. Biol.* **248**, 307–318 (2002). 12167406 10.1006/dbio.2002.0711

[42] Ellett, F., Pase, L., Hayman, J. W., Andrianopoulos, A. & Lieschke, G. J. mpeg1 promoter transgenes direct macrophage-lineage expression in zebrafish. *Blood* **117**, e49-56 (2011). 21084707 10.1182/blood-2010-10-314120 PMC3056479

[43] Davison, J. M. et al. Transactivation from Gal4-VP16 transgenic insertions for tissue-specific cell labeling and ablation in zebrafish. *Dev. Biol.* **304**, 811–824 (2007). 17335798 10.1016/j.ydbio.2007.01.033 PMC3470427

[44] Renshaw, S. A. et al. A transgenic zebrafish model of neutrophilic inflammation. *Blood* **108**, 3976–3978 (2006). 16926288 10.1182/blood-2006-05-024075

[45] Yoo, S. K. et al. Differential regulation of protrusion and polarity by PI3K during neutrophil motility in live zebrafish. *Dev. Cell* **18**, 226–236 (2010). 20159593 10.1016/j.devcel.2009.11.015 PMC2824622

[46] Hall, C., Flores, M. V., Storm, T., Crosier, K. & Crosier, P. The zebrafish lysozyme C promoter drives myeloid-specific expression in transgenic fish. *BMC Dev. Biol.* **7**, 42 (2007). 17477879 10.1186/1471-213X-7-42 PMC1877083

[47] Traver, D. et al. Transplantation and in vivo imaging of multilineage engraftment in zebrafish bloodless mutants. *Nat. Immunol.* **4**, 1238–1246 (2003). 14608381 10.1038/ni1007

[48] Harvie, E. A., Green, J. M., Neely, M. N. & Huttenlocher, A. Innate immune response to *Streptococcus iniae* infection in zebrafish larvae. *Infect. Immun.* **81**, 110–121 (2013). 23090960 10.1128/IAI.00642-12 PMC3536132

[49] Huang, D. W., Sherman, B. T. & Lempicki, R. A. Bioinformatics enrichment tools: paths toward the comprehensive functional analysis of large gene lists. *Nucleic Acids Res.* **37**, 1–13 (2009). 19033363 10.1093/nar/gkn923 PMC2615629

[50] Kanehisa, M. & Goto, S. KEGG: Kyoto encyclopedia of genes and genomes. *Nucleic Acids Res.* **28**, 27–30 (2000). 10592173 10.1093/nar/28.1.27 PMC102409

[51] Kanehisa, M., Furumichi, M., Sato, Y., Matsuura, Y. & Ishiguro-Watanabe, M. KEGG: Biological systems database as a model of the real world. *Nucleic Acids Res.* **53**, D672–D677 (2025). 39417505 10.1093/nar/gkae909 PMC11701520

[52] Paffett-Lugassy, N. N. & Zon, L. I. Analysis of hematopoietic development in the zebrafish. *Methods Mol. Med.* **105**, 171–198 (2005). 15492396 10.1385/1-59259-826-9:171

[53] Chu, C.-Y. et al. The zebrafish erythropoietin: Functional identification and biochemical characterization. *FEBS Lett.* **581**, 4265–4271 (2007). 17706649 10.1016/j.febslet.2007.07.073

[54] Thisse, C. & Thisse, B. High-resolution in situ hybridization to whole-mount zebrafish embryos. *Nat. Protoc.* **3**, 59–69 (2008). 18193022 10.1038/nprot.2007.514

[55] Nakajima, H. et al. Tissue-restricted expression of Nrf2 and its target genes in zebrafish with gene-specific variations in the induction profiles. *PLoS ONE* **6**, e26884 (2011). 22046393 10.1371/journal.pone.0026884 PMC3201981

[56] Fuse, Y., Nakajima, H., Nakajima-Takagi, Y., Nakajima, O. & Kobayashi, M. Heme-mediated inhibition of Bach1 regulates the liver specificity and transience of the Nrf2-dependent induction of zebrafish heme oxygenase 1. *Genes Cells* **20**, 590–600 (2015). 25982796 10.1111/gtc.12249

[57] Paredes-Zúñiga, S. et al. CXCL12a/CXCR4b acts to retain neutrophils in caudal hematopoietic tissue and to antagonize recruitment to an injury site in the zebrafish larva. *Immunogenetics* **69**, 341–349 (2017). 28220184 10.1007/s00251-017-0975-9

