# Supplementary material for: Heme oxygenase 1 activity mediates red blood cell clearance and tail fin regeneration in zebrafish larvae
Source: Sci Rep. 2026 Jun 2;16:17157. doi: 10.1038/s41598-026-54996-x (PMC13234416; doi:10.1038/s41598-026-54996-x)
Supplement: Supplementary file 1 — Supplementary Information 1 [file 41598_2026_54996_MOESM1_ESM.pdf]

Supplementary Figure S1

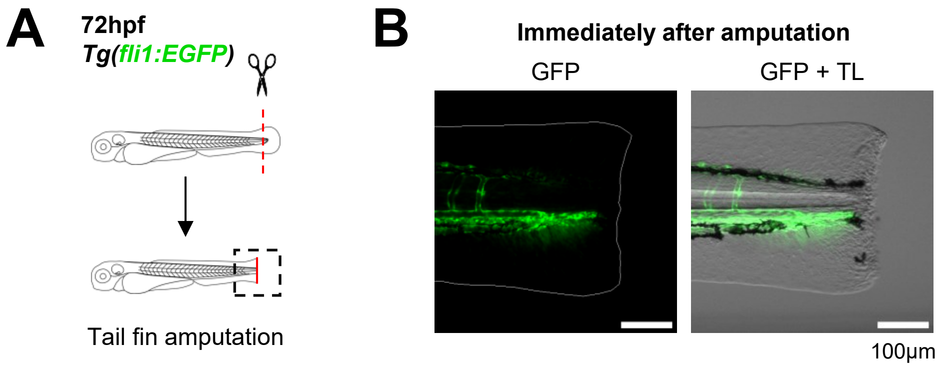

Supplementary Figure S1. Tail fin amputation in zebrafish larva. (**A**) Schematic for experimental design. (**B**) Representative image of tail fin amputated *Tg(fli1:EGFP)* larva, showing that no blood vessels were affected by the injury performed. Scale bar = 200μm.

Supplementary Figure S2

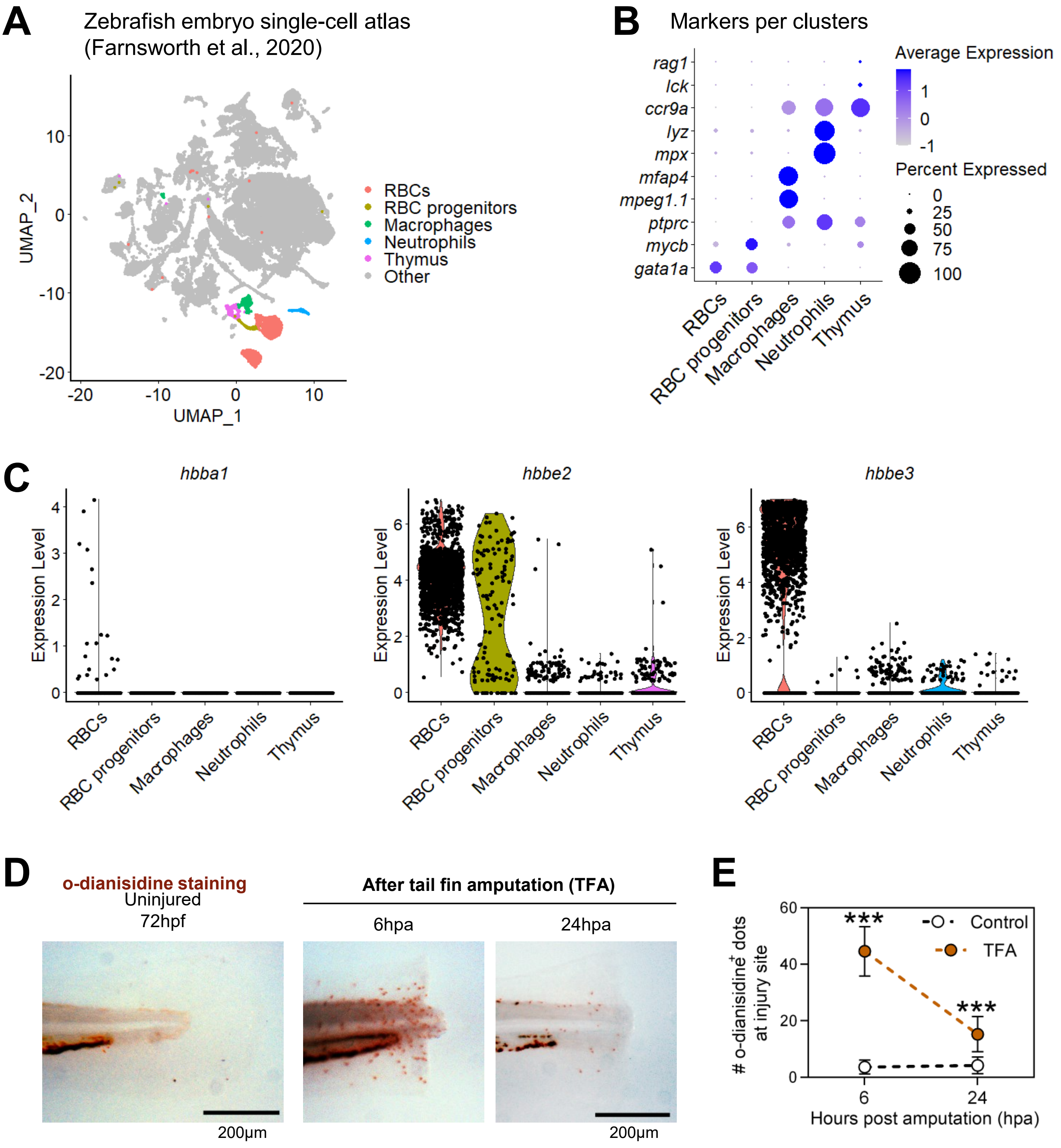

Supplementary Figure S2. Single-cell expression analysis of coding genes for globin proteins identified through proteomics. (**A**) UMAP from the zebrafish embryo single-cell atlas (Farnsworth et al., 2020) showing the distribution of hematopoietic and immune cell clusters. (**B**) Markers per studied cluster. (**C**) Volcano plot showing expression of the hemoglobin genes *hbba1*, *hbbe2*, and *hbbe3* by hematopoietic and immune clusters. (**D**) Representative pictures of control and amputated tail fins of zebrafish larvae stained with o-dianisidine. Scale bar = 200µm. (**E**) Quantification of o-dianisidine^{+} dots in the injury site of tail fin amputated larvae versus time-matched non-amputated controls (mean ± SD; n = 23-24 larvae per condition/timepoint).

Unpaired t-tests were performed in E at the indicated timepoints. ***p<0.001.

Supplementary Figure S3

A RT-PCR from sorted cells

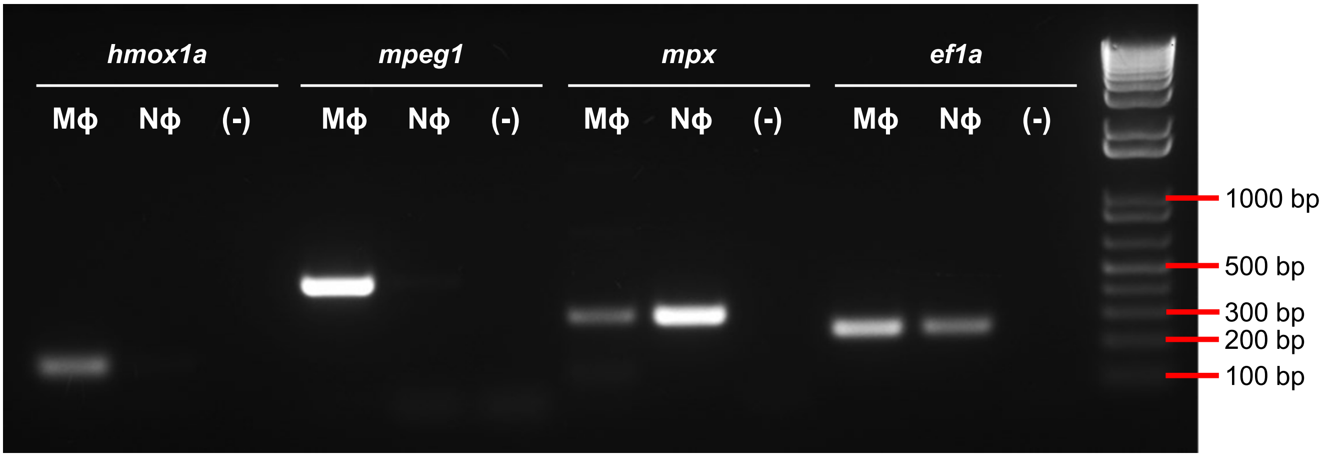

B PCR product size

Gene	*hmxo1a*	*mpeg1*	*mpx*	*ef1a*
**Size (bp)**	158	460	311	257

Supplementary Figure S3. RT-PCR analysis of sorted zebrafish neutrophils and macrophages at 72 hours post fertilization. (**A**) Raw gel image corresponding to Fig. 2B. Specific DNA ladder bands between 100-1000bp are indicated (ladder used was 1kb plus DNA ladder from Invitrogen, Cat. #10787-018). Mϕ = RNA from sorted macrophages, Nϕ = RNA from sorted neutrophils, (-) = no-template PCR control. (**B**) Expected PCR product sizes for each gene tested, based on primers sequences listed in Table S4.

Supplementary Figure S4

A

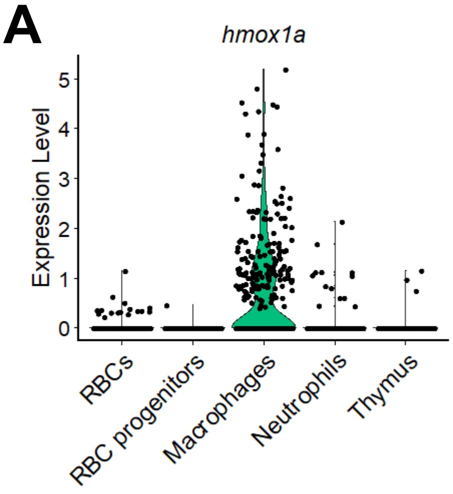

B

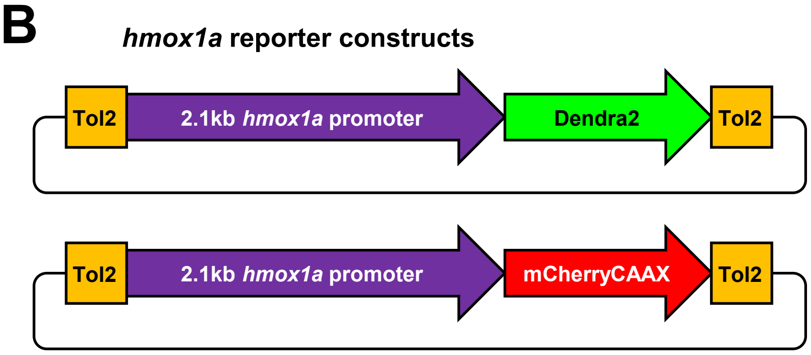

C

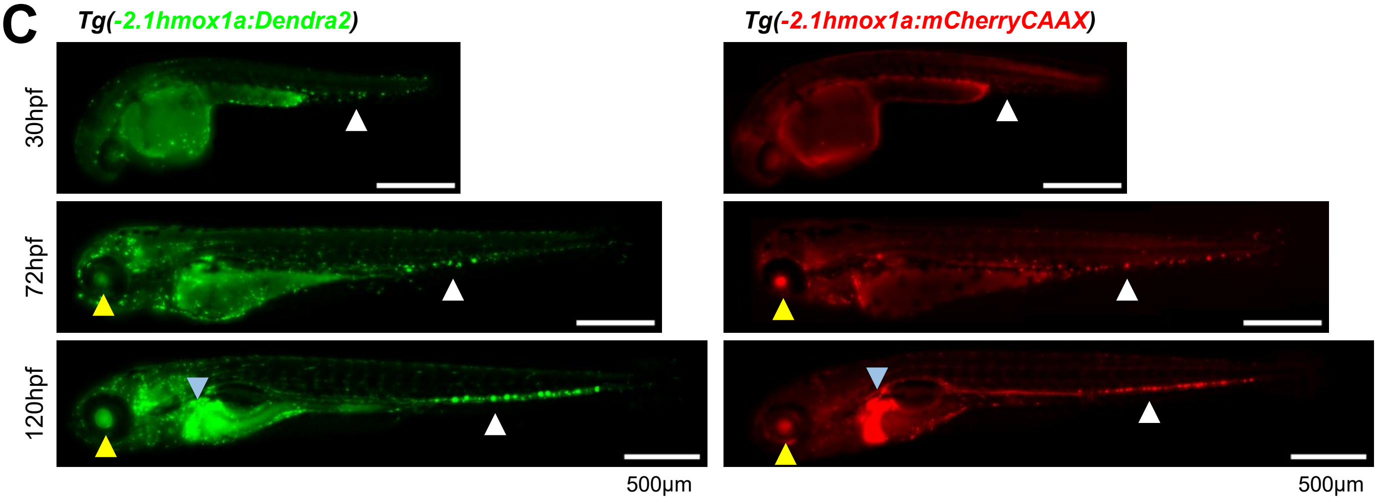

D

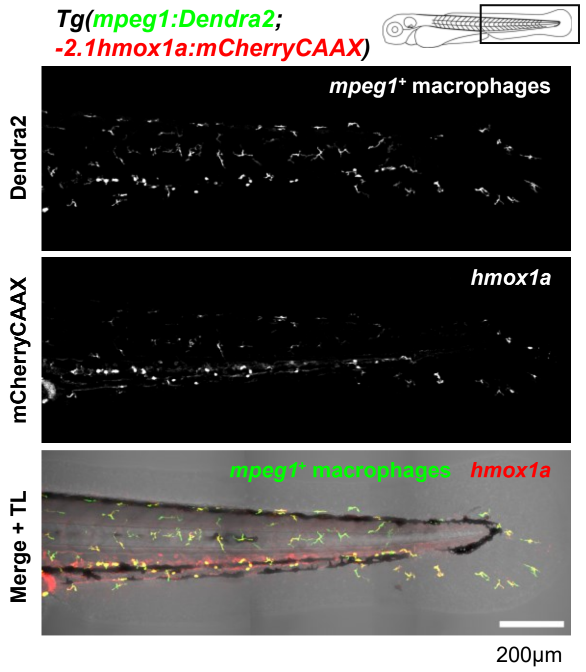

E

Mϕ in tail (n = 6)

	Total Mϕ	*hmox1a*+ Mϕ
# Mϕ	76.33 ± 7.15	62.17 ± 8.91
% tail Mϕ	100%	81.22% ± 5.36%

F

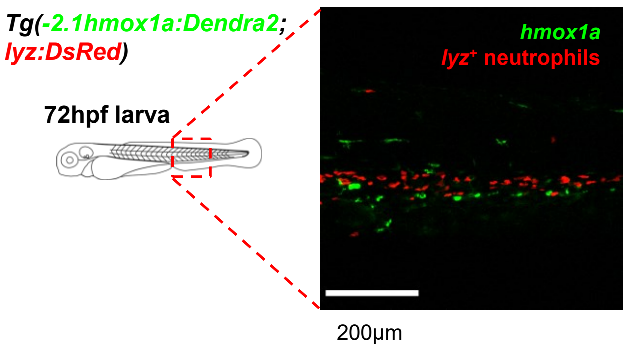

Supplementary Figure S4. The *hmox1a* gene is expressed by zebrafish macrophages. (**A**) Expression of *hmox1a* by hematopoietic and immune clusters from the zebrafish embryo single-cell atlas. (**B**) Maps of the Tol2-based *-2.1hmox1a:Dendra2* and *-2.1hmox1a:mCherryCAAX* plasmids used for transgenesis in zebrafish. (**C**) Fluorescence expression pattern of the generated *hmox1a* transgenic reporters at the indicated timepoints. White arrowheads indicate expression in the rostral blood island/caudal hematopoietic tissue, yellow arrowheads indicate fluorescence in the retina, and sky-blue arrowheads show expression in the liver. Scale bar = 500µm (**D**) Representative picture of the tail of 72hpf *Tg(mpeg1:Dendra2; -2.1hmxo1a:mCherryCAAX)* double transgenic zebrafish larvae. (**E**) Quantification of tail macrophages positive and negative for *hmox1a*. (**F**) Representative image from a tail section of *Tg(-2.1hmox1a:Dendra2; lyz:DsRed)* double transgenic zebrafish larva at 72hpf. Scale bar = 200µm.

Supplementary Figure S5

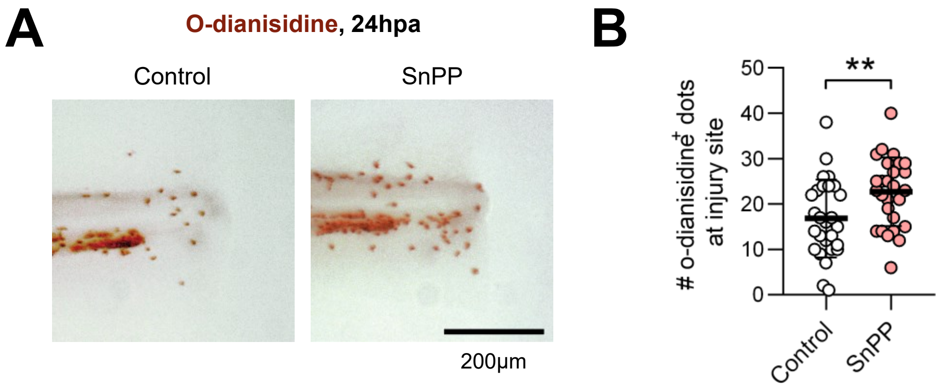

Supplementary Figure S5. Pharmacological inhibition of Hmox1 impairs clearance of o-dianisidine dots from the injury site. (**A**) Representative o-dianisidine staining images of SnPP-treated amputated larvae at 24hpa. Scale bar = 200μm. (**B**) Quantification of o-dianisidine^{+} dots in the injury site of SnPP-treated larvae at 24hpa (1 dot = 1 larva, 27-28 larvae per condition).

Unpaired t-test was performed in B. **p<0.01.

Supplementary Figure S6

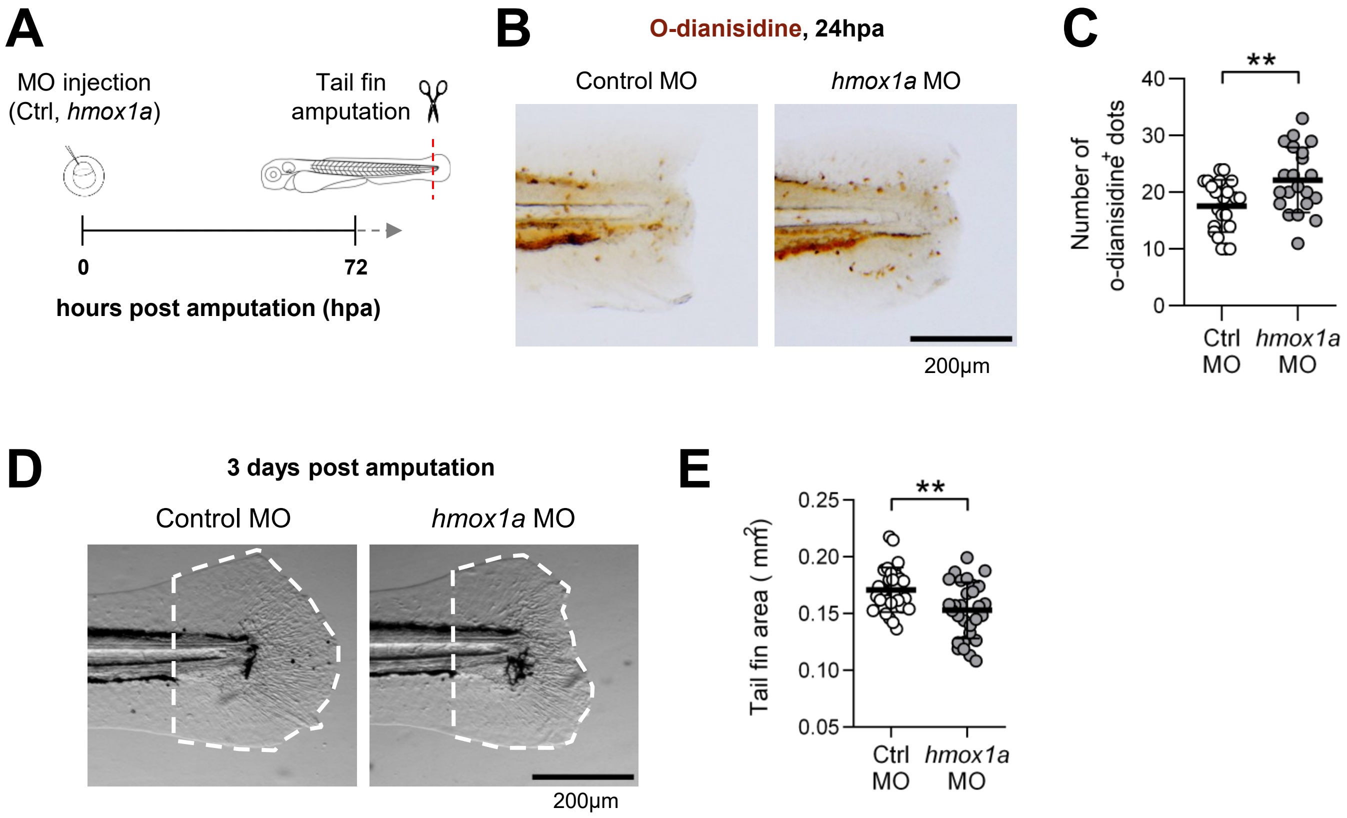

Supplementary Figure S6. Morpholino-mediated knockdown of *hmox1a* impairs RBC clearance from the injury site and affects tail fin regeneration. (**A**) Experimental design for tail in amputations in *hmox1a* and control morphants (**B**) Representative o-dianisidine staining images of amputated *hmox1a* morphants (*hmox1a* MO) and control morphants (Control MO) at 24hpa. Scale bar = 200µm. (**C**) Quantification of o-dianisidine^{+} dots in the injury site of control MO and *hmox1a* MO larvae at 24hpa (1 dot = 1 larva, 27-28 larvae per condition). (**D**) Representative tail fins of amputated control MO and *hmox1a* MO at 3 days post-amputation (3dpa). Scale bar = 200µm. (**E**) Quantification of the tail fin area of control MO and *hmox1a* MO larvae at 3dpa (1 dot = 1 larva, 30 larvae per condition).

Unpaired t-tests were performed in C and E. **p<0.01.

Supplementary Figure S7

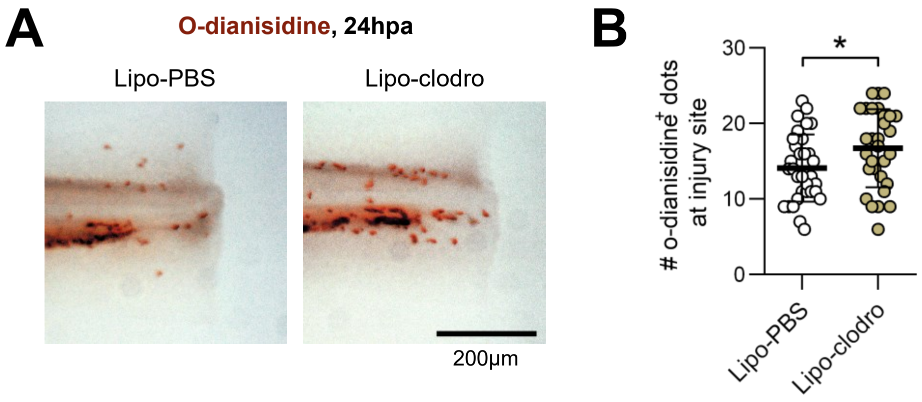

Supplementary Figure S7. Depletion of macrophages impairs clearance of o-dianisidine dots from the injury site. (**A**) Representative o-dianisidine stainings of amputated Lipo-PBS and Lipo-clodro larvae at 24hpa. Scale bar = 200µm. (**B**) Quantification of o-dianisidine$^+$ dots in the injury site at 24hpa (1 dot = 1 larva, 32-34 larvae per condition).

Unpaired t-test was performed in B. *$p<0.05$.

Supplementary Video legends

Supplementary Video 1 (related to Figure 3). Complete time-lapse imaging of tail fin-amputated *Tg(mpeg1:Dendra2; -2.1hmox1a:mCherryCAAX)* larva from 30 minutes until 10 hours post-amputation (hpa), acquired at 5-minute intervals. Amputation is indicated with a white dashed line. Speed = 8 frames/second. Scale bar = 100μm.

Supplementary Video 2 (related to Figure 5). Complete time-lapse imaging of tail fin-amputated *Tg(mpeg1:Dendra2; gata1a:DsRed)* larva from 6 until 10 hours post amputation (hpa), acquired at 3-minute intervals. Amputation is indicated with a white dashed line. Speed = 5 frames/second Scale bar = 100μm.

Supplementary Table legends

Supplementary Table S1. Proteomic profile of tail portions from control and tail fin amputated zebrafish embryos.

Supplementary Table S2. Venn diagram analyses of identified protein-coding genes at inflammatory and resolution phases following tail fin amputation.

Supplementary Table S3. DAVID functional classification of protein-coding genes exclusively expressed in amputated larvae during inflammation and resolution phases.

Supplementary Table S4. List of primers used in this study.
