# Supplementary material for: Heme oxygenase 1 activity mediates red blood cell clearance and tail fin regeneration in zebrafish larvae
Source: Sci Rep. 2026 Jun 2;16:17157. doi: 10.1038/s41598-026-54996-x (PMC13234416; doi:10.1038/s41598-026-54996-x)
Supplement: Supplementary file 5 — Supplementary Information 5 [file 41598_2026_54996_MOESM5_ESM.pdf]

Table S4. List of primers. Restriction enzyme sites included in primers are shown in red.

Cloning of *hmox1a* promoter

Primer set	Forward primer (5' – 3')	Reverse primer (5' – 3')
p-*hmox1a* set 1	CTTTTGACGTCACTAAAGAATGTACG	AATTGAAACTCCTGTAGCTCAC
p-*hmox1a* set 2	CTCGAGCTTTTGACGTCACTAAAGAATGT	CCCGGGGAGTCCATCTCTAAAAAAACAGA

Whole-mount *in situ* hybridization

Primer set	Forward primer (5' – 3')	Reverse primer (5' – 3')
hmox1b ISH	GGGGATCCATGCTGAGCTACCAGAGGG	GGCTCGAGTCTCAACAGTACAAATGTGCCG

RT-PCR gene expression analyses

Primer set	Forward primer (5' – 3')	Reverse primer (5' – 3')
eef1a1l1 (ef1a)	TTCTGTTACCTGGCAAAGGG	TTCAGTTTGTCCAACACCCA
hmox1a	GATGCTCAGCTACCAGAAAGGACA	TCTGGCCAATTCCTGAGGGAAGTA
mpeg1.1	GCGATCATGAAGTCAAGAGCA	GGACTTGAACCCGTGCTGTA
mpx	GCTGCTGTTGTGCTCTTTCA	TTGAGTGAGCAGGTTTGTGG
